# Selenium Nanodots (SENDs) as Antioxidants and Antioxidant‐Prodrugs to Rescue Islet *β* Cells in Type 2 Diabetes Mellitus by Restoring Mitophagy and Alleviating Endoplasmic Reticulum Stress

**DOI:** 10.1002/advs.202300880

**Published:** 2023-04-21

**Authors:** Qiong Huang, Zerun Liu, Yunrong Yang, Yuqi Yang, Ting Huang, Ying Hong, Jinping Zhang, Qiaohui Chen, Tianjiao Zhao, Zuoxiu Xiao, Xuejun Gong, Yitian Jiang, Jiang Peng, Yayun Nan, Kelong Ai

**Affiliations:** ^1^ Department of Pharmacy Xiangya Hospital Central South University Changsha 410008 China; ^2^ National Clinical Research Center for Geriatric Disorders Xiangya Hospital Central South University Changsha 410008 China; ^3^ Xiangya School of Pharmaceutical Sciences Central South University Changsha 410078 China; ^4^ Hunan Provincial Key Laboratory of Cardiovascular Research Xiangya School of Pharmaceutical Sciences Central South University Changsha 410078 China; ^5^ Pancreatic Surgery Xiangya Hospital Central South University Changsha 410008 China; ^6^ Geriatric Medical Center People's Hospital of Ningxia Hui Autonomous Region Yinchuan 750002 China

**Keywords:** endoplasmic reticulum stress, GPX1, mitophagy, prodrug, ROS scavenger, selenium nanodots, Type 2 diabetes mellitus

## Abstract

Preventing islet *β*‐cells death is crucial for treating type 2 diabetes mellitus (T2DM). Currently, clinical drugs are being developed to improve the quality of T2DM care and self‐care, but drugs focused on reducing islets *β*‐cell death are lacking. Given that *β*‐cell death in T2DM is dominated ultimately by excessive reactive oxygen species (ROS), eliminating excessive ROS in *β*‐cells is a highly promising therapeutic strategy. Nevertheless, no antioxidants have been approved for T2DM therapy because most of them cannot meet the long‐term and stable elimination of ROS in *β*‐cells without eliciting toxic side‐effects. Here, it is proposed to restore the endogenous antioxidant capacity of *β*‐cells to efficiently prevent *β*‐cell death using selenium nanodots (SENDs), a prodrug of the antioxidant enzyme glutathione peroxidase 1 (GPX1). SENDs not only scavenge ROS effectively, but also “send” selenium precisely to *β*‐cells with ROS response to greatly enhance the antioxidant capacity of *β*‐cells by increasing GPX1 expression. Therefore, SENDs greatly rescue *β*‐cells by restoring mitophagy and alleviating endoplasmic reticulum stress (ERS), and demonstrate much stronger efficacy than the first‐line drug metformin for T2DM treatment. Overall, this strategy highlights the great clinical application prospects of SENDs, offering a paradigm for an antioxidant enzyme prodrug for T2DM treatment.

## Graphical abstract

Selenium nanodots (SENDs) are obtained by dehydration polymerization of selenocysteine under alkaline conditions. SENDs can effectively restore and enhance the antioxidant capacity of islet *β* cells to greatly improve mitophagy, restore mitochondrial dysfunction, reduce endoplasmic reticulum stress (ERS) and, finally, rescue islet *β* cells in type 2 diabetes mellitus.

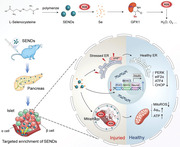

## 1 Introduction

Currently type 2 diabetes mellitus (T2DM) affects 437 million people worldwide,^[^ ^1^ ^]^ especially older (age >65 years) people, who account for nearly half of all adults diagnosed with diabetes.^[^ ^2^ ^]^ T2DM increases the risk of cardiovascular disease, kidney disease, liver disease, cancer, and infection substantially.^[^ ^3^ ^]^ The risk of all‐cause mortality in T2DM is 1.8‐times that people not suffering from T2DM, and the medical costs of T2DM have been reported to be 2.3‐times those of non‐T2DM.^[^ ^4^ ^]^ The main feature of T2DM is hyperglycemia, which is caused by damage to islet *β* cells and insulin resistance.^[^ ^5^ ^]^ Of particular importance, islet *β* cells damage is the most crucial factors of T2DM. Compared with nondiabetic people, the number of islet *β* cells is reduced by ≈50% in T2DM patients.^[^ ^6^ ^]^ Currently, >10 classes of drugs have been approved for T2DM treatment, such as biguanides, sulfonylureas, meglitinides, amylin mimetics, glucagon‐like peptide‐1 analogs, and insulin.^[^ ^7^ ^]^ These drugs have contributed to dramatic improvements in the quality of diabetes care and self‐care in recent years, but there are still no drugs for the underlying pathological process leading to the destruction of islet *β* cells, which is what ultimately leads to the onset and progression of T2DM.^[^ ^8^ ^]^

Emerging evidence has revealed that islet *β* cell death in T2DM is closely related to (and ultimately dominated by) excessive reactive oxygen species (ROS) levels.^[^ ^9^ ^]^ High levels of ROS in islet *β* cells are mediated mainly by hyperglycemia and chronic inflammation.^[^ ^10^ ^]^ When abnormally high blood glucose and fluctuations persist for long periods of time, glucose metabolism is diverted to other pathways to produce more ROS, including methylglyoxal/glycation, enediol formation, activation of diacylglycerol/protein kinase C, hexosamine metabolism, and sorbitol metabolism.^[^ ^11^ ^]^ All of these additional pathways generate excessive ROS in islet *β* cells. Chronic inflammation is another prominent feature of T2DM.^[^ ^12^ ^]^ Hyperglycemia, amylin polypeptide, free fatty acids, and mitochondrial ROS (mtROS) mediate the production of proinflammatory cytokines such as interleukin (IL)‐1*β*, and tumor necrosis factor‐*α*. The islet *β* cells are highly responsive to inflammatory factors.^[^ ^13^ ^]^ For example, the islet *β* cells have much higher expression of the IL‐1 receptor than all other cell types. Moreover, inflammatory responses due to cytokines (e.g., IL‐1) persist longer and do not oscillate in islet *β* cells, and eventually generate a “ROS storm” by activating the reduced form of nicotinamide adenine dinucleotide phosphate (NADPH) oxidase and inducible nitric oxide synthase.^[^ ^8^ ^]^ On the other hand, islet *β* cells inherently have low antioxidant capacity based on the need for insulin secretion,^[^ ^10^ ^]^ which makes islet *β* cells highly vulnerable to high levels of ROS, thereby leading to mitochondrial dysfunction, endoplasmic reticulum stress (ERS), insufficient insulin secretion, and eventual death. These evidences fully demonstrate that ROS are crucial therapeutic targets to rescue *β* cells. Nevertheless, currently no antioxidants have been approved for T2DM treatment, mainly because of a series of deficiencies in conventional antioxidants, such as low bioavailability, weak targeting of *β* cells, and strong side‐effects. Moreover, T2DM is a long‐term, progressive chronic disease. Therefore, to eliminate the high levels of ROS in *β* cells effectively, one must also necessary to eliminate ROS in islet *β* cells for a long time. However, few antioxidant drugs available currently meet such stringent requirements.^[^ ^14^ ^]^

**Figure 1:**
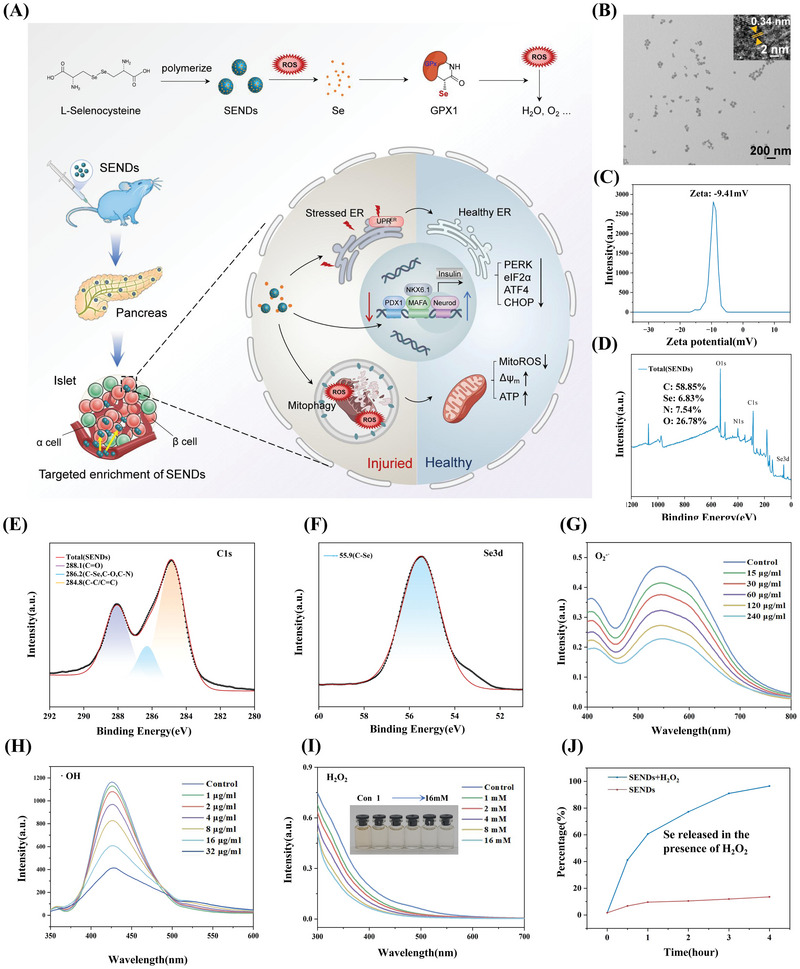
Synthesis and characterization of SENDs. A) Schematic illustration of preparation and treatment effect of SENDs. B) TEM image of SENDs. Scale bar: 200 nm. Inset: enlarged image of SENDs. Scale bar: 2 nm. C) Zeta potential distribution intensity of SENDs. D–F) XPS spectrum of SENDs D), C1s E), and Se3d F) in SENDs. G–I) O_2_^·−^ G), ·OH H), and H_2_O_2_ I) scavenging ability of different concentrations of SENDs. J) Release degree of Se in SENDs with or without H_2_O_2_.

Here, for the first time, we developed SENDs aimed to restore and enhance the antioxidant capacity of islet *β* cells to efficiently avoid their destruction in T2DM. Thanks to the rich blood supply of the pancreas, fenestrated capillaries surrounding *β* cells in islets,^[^ ^15^ ^]^ and the increased permeability of islet vascular endothelium caused by chronic inflammation in T2DM,^[^ ^16^ ^]^ SENDs could be enriched in islets and enter *β* cells to eliminate high levels of ROS after intravenous injection. More importantly, SENDs are prodrugs of the endogenous and highly effective antioxidant selenoenzyme glutathione peroxidase 1(GPX1) in islet *β* cells. SENDs “send” selenium (Se; the key factor of GPX1) to islet *β* cells by releasing Se under high levels of ROS to promote GPX1 expression in islet *β* cells. Low selenium intake is also associated with the increased prevalence of T2DM.^[^ ^17^ ^]^ Therefore, SENDs could effectively restore and enhance the antioxidant capacity of islet *β* cells to greatly improve mitophagy, restore mitochondrial dysfunction, reduce ERS and, finally, rescue islet *β* cells in T2DM (**Figure** **1**A). After SENDs treatment, not only the blood glucose concentration and insulin secretion index were greatly restored, but more importantly, the death of *β* cells was greatly inhibited in T2DM. The therapeutic effect of SENDs was also higher than that of metformin (first‐line drug for clinical T2DM) and the number of *β* cells was 3‐times that of the metformin treatment group. In addition, SENDs contained only the elements necessary for human body and had excellent biocompatibility. This work may provide valuable guidance for T2DM treatment and fundamentally solve the problem of islet *β* cell death in T2DM.

## 2 Results

### 2.1 Synthesis and Characterization of SENDs

SENDs were obtained by dehydration polymerization of selenocysteine under alkaline conditions (Figure 1A). As shown by transmission electron microscopy (TEM) and the zeta potential, SENDs had monodispersed properties with a mean diameter of ≈30 nm (Figure 1B) and carried a negative charge (Figure 1C). Such properties allow SENDs to be enriched efficiently in pancreatic tissue through the blood circulation system because of the abundant blood supply to the pancreas and the increased vascular permeability of the islet lesioned site in T2DM. A combination of high‐resolution TEM (Figure 1B, inset) and the unique diffraction hump at 26° in X‐ray diffraction (XRD) results (Figure S1, Supporting Information) suggested that the SENDs had a graphite sheet structures with a 0.34 nm spacing of the graphite crystal plane (002). As shown in Figure 1D, the main component of SENDs was elemental C (58.85%), and the content of Se element in SENDs was also as high as 7.54% according to X‐ray photoelectron spectroscopy (XPS). The C1s XPS fine peak indicated the presence of abundant carboxyl and phenolic hydroxyl groups (C=O and C—O bonds) in SENDs, which explained why SENDs presented a negative charge in aqueous solution (Figure 1E). More importantly, the Se peak at 55.9 was completely attributed to the C—Se bond through the analyses of the XPS fine peak of Se3d, indicating that Se was embedded into the carbon skeleton by a covalent bond (Figure 1F). Se itself can be oxidized readily upon being dissolved in water,^[^ ^18^ ^]^ while this unique structure of Se in SENDs effectively ensures its stability under physiological conditions. In addition, SENDs had abundant N—H groups according to the N1s XPS fine spectrum (Figure S2, Supporting Information), and could be functionalized readily through amide bonds. Meanwhile, as shown in Figure 1E (C1s XPS fine peak) and Figure S3 (Supporting Information) (O1s XPS fine peak), SENDs contained abundant phenolic and carbonic groups. These groups and C—Se groups in SENDs were reductive and nucleophilic, which could eliminate oxidative and electrophilic ROS effectively. O_2_^−·^, ·OH and H_2_O_2_, as three important ROS in T2DM, were adopted to verify the ability of SENDs to eliminate ROS. As shown in Figure 1G, SENDs could eliminate O_2_^−·^ effectively in a concentration‐dependent manner with a superoxide dismutase (SOD)‐like activity of 4.51 U mg^−1^. Moreover, SENDs could eliminate 90% of ·OH even at a concentration as low as 32 µg mL^−1^ (Figure 1H). In contrast to the unstable O_2_^−·^and ·OH, H_2_O_2_ can exist stably and across the membrane freely. H_2_O_2_ can cause oxidative stress damage directly, but also increase the expression of proinflammatory proteins to induce islet *β* cells to produce more ROS.^[^ ^19^ ^]^ Fortunately, SENDs could react efficiently with H_2_O_2_ (Figure 1I), and the color of SENDs faded gradually with an increase in the H_2_O_2_ concentration (Figure 1I, inset), suggesting that the Se element in SENDs might be released into the solution as a colorless Se ion thanks to the “etching” of C—Se bonds by H_2_O_2_. To verify this process, inductively coupled plasma‐mass spectrometry (ICP‐MS) was adopted to measure the content of Se ions in the H_2_O_2_ and SENDs reaction system. As shown in Figure 1J, SENDs remained stable with only 13.5% of Se being released in 4 days under physiological conditions, because Se was embedded firmly in the carbon skeleton of SENDs. Once H_2_O_2_ was present, Se was released from SENDs continuously and slowly, and Se release reached 96.5% in 4 days. Importantly, this strategy of releasing soluble Se slowly could avoid the tissue damage caused by excessive Se ions, and allow Se to be absorbed by islet *β* cells for conversion into GPX1 to recover their own ROS‐scavenging ability.

### 2.2 Distribution of SENDs in T2DM

**Figure 2:**
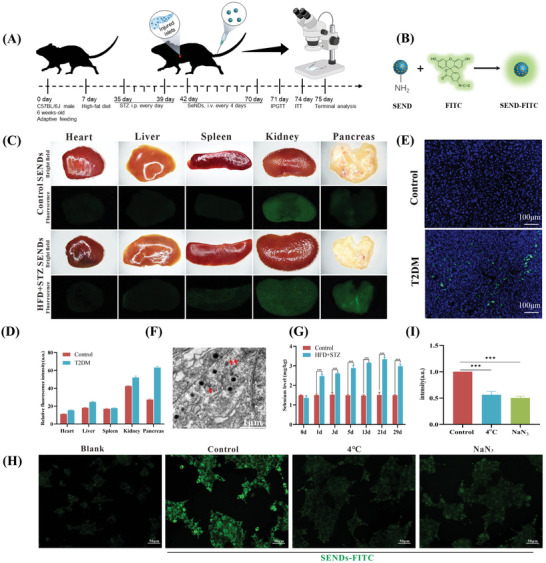
Distribution of SENDs in T2DM. A) Schematic illustration of the establishment and treatment schedule of T2DM mice. B) Synthesis of FTIC‐SENDs: modification of FTIC on aminated SENDs. C) In vivo distributed stereoscopic fluorescence of SENDs. (D) Quantification of in vivo distributed fluorescence intensity of C). E) Fluorescent staining of frozen pancreas sections for SENDs. Scale bar: 100 µm. F) TEM image showing SENDs (red arrow) in islet *β* cells. Scale bar: 1 µm. G) Quantification of the Se content in pancreatic tissues after the treatment determined by ICP‐MS. H,I) Fluorescence images H) and quantification I) of INS‐1 cells after incubation with SENDs under different conditions as indicated. Scale bar: 50 µm. Data represent means ± S.D. from three independent replicates. (***p < 0.001 vs Control group).

Whether SENDs reach the islet *β* cells accurately is crucial to ensure their efficacy and safety in T2DM. Male C57 BL/6 mice (8 weeks) were fed adaptively with normal chow for 1 week, fed a high‐fat diet (HFD) continuously for 1 month, and then given streptozotocin (STZ; 60 mg kg^−1^, i.p., once‐daily) for 5 days to construct a T2DM model (**Figure** **2**A). A fasting blood glucose level (FBGL) ≥11.1 mm was considered to denote successful modeling. To verify the targeting effect of SENDs, SENDs were attached to the fluorescein isothiocyanate (FITC; a strong fluorescent group) via NH_2_ groups, injected into T2DM mice via the tail vein, and tracked in vivo by stereo fluorescence microscopy (Figure 2B; and Figure S4, Supporting Information). As shown in Figure 2C,D, SENDs could effectively target the pancreas in T2DM mice, and their distribution was much higher than that of the normal pancreas and other important organs in mice. In healthy mice, the distribution of SENDs was high in the kidney and low in the heart, liver, spleen, and pancreas, whose uniformly low fluorescence intensity was derived from the low residual of SENDs. In T2DM mice, the pancreas was greatly damaged, resulting in an enlarged endothelial cell gap due to damage to the capillary endothelium of the islets, which promoted the distribution of SENDs in the pancreas. As shown in Figure S5 (Supporting Information), the capillary endothelial gap of islet tissue of diabetic mice was much larger than that of healthy mice. The specificity of the distribution of SENDs was reconfirmed by the bright green fluorescence in cryosections of diabetic pancreas (Figure 2E). The islets are surrounded by a glomerular like network of fenestrated capillaries (constitute 8–10% of the volume of islets) and have a rich blood supply.^[^ ^20^ ^]^ Meanwhile, the advanced glycosylation end products (AGEs) induce ROS generation directly in T2DM, cause structural damage to the vascular endothelium in islets, and finally disrupt the integrity of the vascular wall to increase vascular permeability for nanomedicines.^[^ ^21^ ^]^ These physiological and pathological features of islets provided a promising therapeutic opportunity for SENDs to target *β* cells through the window pore. As expected, SENDs could be visually observed to enter *β* cells in T2DM, whose prominent feature was to contain spherical insulin granules (Figure 2F). Moreover, the pancreas of T2DM mice showed a higher and stabler Se level compared with that in healthy groups according to ICP‐MS after SENDs injection (Figure 2G). These evidences demonstrated that SENDs targeted *β* cells efficiently in T2DM. Interestingly, SENDs were also relatively highly enriched in the kidneys of healthy mice and T2DM mice, suggesting that they could be excreted from the body through the urinary system (Figure 2C,D). We further measured Se content in vital organs by ICP. Similar to stereoscopic fluorescence, Se content remained normal in vital organs (Figure S6A, Supporting Information), while its distribution in the kidneys was higher on the first day and normalized on the fourth day (Figure S6B, Supporting Information). SENDs have a graphene‐like structure (derived from a conjugated carbon plane), and can pass through the glomerular filtration system by rolling, curling, and folding under the pressure of blood flow, though the size of SENDs is slightly larger than that of the glomerular filtration barrier.^[^ ^22^ ^]^ This feature allowed SENDs to be excreted to prevent their excessive accumulation in vivo and greatly increased the biocompatibility of SENDs. How SENDs enter islet *β* cells was also explored further. As shown in Figure 2H,I, SENDs could enter the rat islet cell tumor cells (INS‐1) efficiently when SENDs and INS‐1 cells were coincubated. The mean fluorescence intensity decreased to 56.3% and 50.3% from that of normal conditions in the low‐ATP environment caused by low temperature or NaN_3_, respectively, suggesting that the entry of SENDs into INS‐1 cells required energy support, and was an active‐transport process.

### 2.3 The Therapeutic Effect of SENDs in T2DM

**Figure 3:**
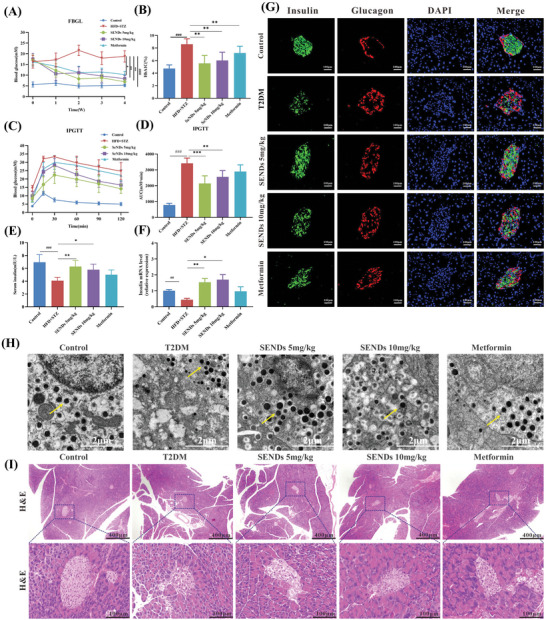
The therapeutic effect of SENDs in T2DM. A) The FBGL of mice during the drug treatment. B) The percentage of serum HbA1C from each group. C) IPGTT was measured after 3 weeks of drug administration. D) The AUC of IPGTT. E) The serum insulin level from each group. F) The relative mRNA level of insulin in pancreas from each group. G) Immunofluorescent staining of insulin (green), glucagon (red), DAPI (blue), and their merge images of the islet tissue sections from each group. Scale bar: 100 µm. H) TEM images showing insulin granules (yellow arrow) in islet *β* cells from each group. Scale bar: 1 µm. I) Representative images of H&E staining of pancreatic slices in each group. Scale bar: 400, 100 µm from top to bottom. Data represent means ± S.D. from at least five independent replicates. (^##^ *p* < 0.01, ^###^ *p* < 0.001 vs Control group, *p < 0.05, **p < 0.01, ***p < 0.001 vs T2DM group).

Based on the powerful ROS scavenging ability and the sustained targeting to islet *β* cells of SENDs, the therapeutic effects of SENDs were further tested in T2DM mice. T2DM mice were received 5 and 10 mg kg^−1^ SENDs (every 4 days) and 200 mg kg^−1^ metformin (every day) for 4 weeks (Figure 2A). The glycemic control indexes were analyzed and recorded the weekly FBGL during the treatment and glycated hemoglobin (HbA1c) levels at the end of the treatment. FBGL ≥ 7.0 mm or HbA1c ≥ 6.5% indicates the occurrence of T2DM.^[^ ^23^ ^]^ Metformin is a clinically widely used diabetes drug, whose hypoglycemic effect has been fully verified and confirmed. More importantly, metformin may reduce ROS in *β* cells by reducing systemic inflammation.^[^ ^24^ ^]^ As shown in **Figure** **3**A,B, untreated T2DM mice had FBGL above 15 mm and HbA1c above 8%, while both indexes were normalized after SENDs treatment. Remarkably, HbA1c decreased to 5% after two doses of SENDs, which was more effective than metformin (7%). SENDs also improved severe weight loss in T2DM to great extent (Figure S7, Supporting Information). These results indicated that SENDs could well control blood glucose in T2DM mice. To explore how SENDs achieve this effect, the glucose metabolic capacity of mice was further assessed by intraperitoneal glucose tolerance test (IPGTT) on the day after treatment. As shown in Figure 3C, the FBGL in untreated T2DM mice was significantly higher than those in healthy mice during 120 min IPGTT, indicating that glucose tolerance was impaired severely in T2DM. While 5 mg kg^−1^ SENDs almost reversed the glucose tolerance curve back to normal and decreased the IPGTT area under curve (AUC) by 32.8% compared to the T2DM group, which was better than metformin (decreased by 15.3%) (Figure 3D), demonstrated that stabilization of blood glucose levels by SENDs was likely to be associated with stimulation of insulin secretion. The efficacy of conventional Se on T2DM also be studied. As shown in Figure S8 (Supporting Information), conventional Se had a very slight therapeutic effect, which may be attributed to the limited bioavailability of Se by oral administration, especially the difficulty of targeting to islets with high oxidative stress. Sigliptin is a highly selective dipeptidyl peptidase 4 (DPP‐4) inhibitor, and can promote the islet *β* cells to synthesize and release insulin significantly by increasing the level of active incretin in T2DM patients. Interestingly, SENDs could still exhibit a better therapeutic effect than Sigliptin (Figure S8, Supporting Information).

To this end, the T2DM mice were euthanized on day 5 after treatment and the effects of SENDs on islet *β* cells and insulin secretion were analyzed. As expected, both insulin serum contents and mRNA expression levels increased significantly in T2DM mice treated with SENDs in comparison to the weak effect of metformin, especially after treatment with 5 mg kg^−1^ SENDs, which were 1.5‐fold and 3‐fold higher than those in T2DM, respectively (Figure 3E,F). The effect of SENDs was further analyzed with immunofluorescence dual localization of insulin (green) and glucagon (red), both of which were produced by islet *β* cells and *α* cells, respectively. The distribution of red and green fluorescence revealed the structural characteristics of islets, and their count ratios indicated the proportion of islet *β* cells per islet. As shown in Figure 3G, green fluorescence was dominant and central, while red fluorescence was minor and peripheral, which matched the typical distribution of islets. The ratio of *β* cells to *α* cells decreased from 2.27 (in healthy mice) to 0.32 (in T2DM mice), but recovered to 1.88 after 5 mg kg^−1^ SENDs treatment, which was more effective than metformin (1.42) (Figure 3G; and Figure S9, Supporting Information). The appropriate oxidative environment is necessary for insulin secretion in normal islets. During the process of glucose stimulated insulin secretion (GSIS, the oxidative phosphorylation of glucose, which is accompanied by the production of ROS, is necessary for increasing the ratio of ATP/ADP in islet *β* cells. However, excess ROS in T2DM promotes *β*‐cell death, resulting in a much lower number of *β* cells in the diabetic pancreas than in the normal pancreas.^[^ ^6^ ^]^ More importantly, the excess ROS directly damaged the mitochondrial function of *β* cells, which eventually led to the impairment of insulin synthesis and secretion in *β* cells. SENDs treatment significantly restored the mitochondrial function of *β* cells to promote the production of ATP (Figure 6D), which not only reduced the death of *β* cells but also effectively restored the insulin synthesis and secretion of *β* cells in T2DM mice. Correspondingly, SENDs increased the mRNA (Figure 3F) and protein (Figure 3G) levels of insulin. In generally, insulin was not directly secreted outside the cell after being produced by islet *β* cells, but was temporarily encapsulated in intracellular insulin secretory granules and then expelled by exocytosis in response to glucose‐stimulated intracellular Ca^2+^ levels.^[^ ^25^ ^]^ Correspondingly, the number and size of insulin granules also directly reflect the functional status of islet *β* cells. Therefore, the ultrastructure of islet *β* cells were further investigated by TEM of islets tissues. As shown in Figure 3H, T2DM *β* cells had more and larger insulin granules after SENDs treatment, indicating that SENDs enhanced both the number and function of islet *β* cells. After that, hematoxylin‐eosin (H&E) staining of the pancreatic sections were performed to verify the islet repair ability of SENDs from a holistic perspective (Figure 3I). Mice in advanced T2DM had atrophic and malformed islets due to islet decompensation, but these abnormalities were reversed after SENDs treatment, resulting in more regular shapes and normal areas of islets. These evidences strongly suggested that SENDs effectively prevented islet *β* cell death and increase insulin secretion, surpassing the effect of the clinical first‐line drug metformin.

**Figure 4:**
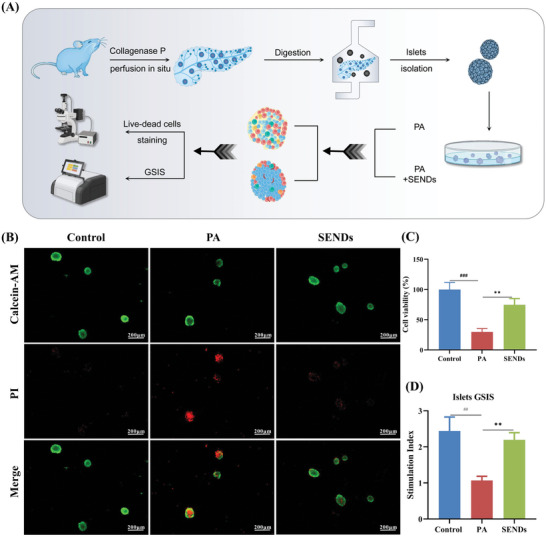
Islet organoids culture and GSIS experiment in vitro. A) Schematic illustration of pancreatic islet‐like organ culture and GSIS experiment. B) Immunofluorescent staining of Calcein‐AM (green) staining, PI (red) staining, and their merge images of live/dead islet *β* cells from each group. Scale bar: 200 µm. C) Cell viability of islet *β* cells from each group. D) Stimulation index of GSIS experiments of pancreatic islet‐like organs from each group. Data represent means ± S.D. from at least three independent replicates. (^##^ *p* < 0.01, ^###^ *p* < 0.001 vs Control group; **p < 0.01, ***p < 0.001 vs PA group).

Considering that pancreatic tissue exocrine glands may interfere with the test results, isolate islets from pancreas and culture islet organoids further to verify the efficacy of SENDs for T2DM treatment (**Figure** **4**A). Palmitic acid (PA; the most abundant saturated fatty acid in the human body) could increase intracellular ROS to reduce islet *β* cells viability, and was used to construct a model of *β* cell injury in T2DM.^[^ ^26^ ^]^ Live and dead islet *β* cells were detected by fluorescence microscopy using Calcein‐AM and propidium iodide as probes, respectively. As shown in Figure 4B,C, islet cell viability decreased to 30.0% after coculture with 0.4 mm PA for 24 h, but SENDs increased it to 81.3%, implying a significant improvement in islet cell death by SENDs. Interestingly, PA‐induced islet cell death showed an inside‐out trend, according with the islet cell structure shown above (Figure 3G), confirming that SENDs could specifically restore islet *β* cells activity once again. GSIS) were further performed on islets in different groups to detect the ability of islet *β* cells to respond to glucose stimulation.^[^ ^27^ ^]^ As shown in Figure 4D, the stimulation index increased directly from 1.0 to 2.2 in the PA group after SENDs treatment, which was almost close to that in the healthy group, indicating that SENDs could well reverse the decrease in *β* cell glycolytic stimulation response capacity caused by PA, which was the same as the results of GSIS experiments based on rat islet cell tumor cells (INS‐1) (Figure S10, Supporting Information). Moreover, stimulation index of islets derived from T2DM mice recovered from 1.14 to 1.82 after SENDs treatment relative to phosphate buffer saline (PBS) (Figure S11, Supporting Information). Given that H_2_O_2_ homeostasis plays an important role in insulin secretion, and high concentration of H_2_O_2_ can damage the GSIS of *β* cells,^[^ ^28^ ^]^ we further investigated the effects of SENDs on GSIS in vitro when INS‐1 cells were cocultured with high level of H_2_O_2_. SENDs could effectively mitigate the reduction of GSIS function in *β* cells caused by high concentrations of H_2_O_2_ (Figure S12, Supporting Information). All above evidence suggested that SENDs had a superior therapeutic effect compared to metformin because SENDs could maintain islet *β* cells functional homeostasis and effectively reverse islet *β* cell death in T2DM.

### 2.4 SENDs Alleviate Oxidative Stress

**Figure 5:**
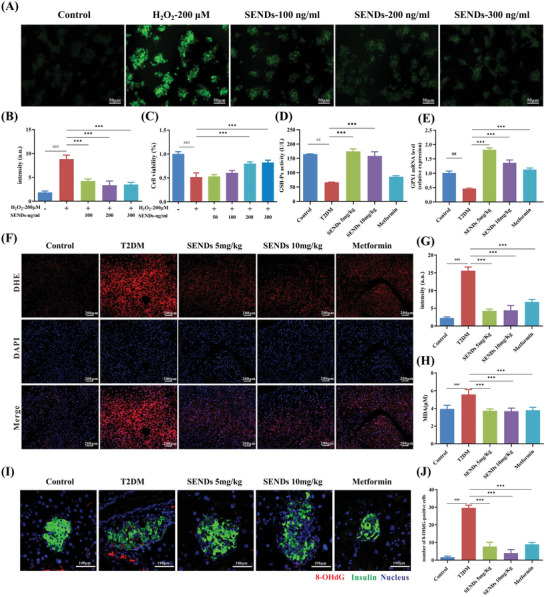
SENDs alleviate oxidative stress. A,B) DCFH‐DA (a ROS probe) staining images of H_2_O_2_‐stimulated INS‐1 cells A) and quantitative results B) under different treatment conditions. Scale bar: 50 µm. C) Cell viability of H_2_O_2_‐stimulated INS‐1 cells treated with different concentrations of SENDs. D) Tissue glutathione peroxidase (GSH‐Px) activity was assessed using a GSH‐Px assay kit. E) The relative mRNA levels of GPX1 in pancreatic tissue homogenates from each group. F) DHE staining (red), DAPI (blue), staining, and their merge images of pancreatic tissues from each group. Scale bar: 200 µm. G) Quantification of fluorescence intensity of DHE. H) MDA levels measured in pancreatic tissue homogenates from each group. I) 8‐OHdG staining (red), insulin (green) and DAPI (blue) merge images of pancreatic tissues from each group. Scale bar: 100 µm. J) Quantification of 8‐OHdG‐positive cells in (I). Data represent means ± S.D. from at least three independent replicates. (^##^ *p* < 0.01, ^###^ *p* < 0.001 vs Control group, ***p < 0.001 vs model group).

The alleviation of oxidative stress in the islet *β* cells by SENDs was the most crucial way to exert their therapeutic effects. Therefore, the ability of SENDs to scavenge ROS was investigated in INS‐1 cells with a 2″,7″‐dichlorodihydrofluorescein diacetate (DCFH‐DA) probe. DCFH‐DA can be oxidized to fluorescent 2″,7″‐dichlorofluorescein (DCF) and emit a green fluorescent signal upon reaction with ROS.^[^ ^29^ ^]^ Strong green fluorescence was observed after stimulation of INS‐1 cells with H_2_O_2_ or PA, and the mean fluorescence intensity was decreased significantly (by 50%) after treatment with SENDs (**Figure** **5**A,B; and Figure S13, Supporting Information). Correspondingly, the activity of INS‐1 cells treated with H_2_O_2_ or PA was recovered significantly as the concentration of SENDs increased. As shown in Figure 5C; and Figure S14 (Supporting Information), cell activity had recovered by ≥80% when the concentration of SENDs was 0.2 µg mL^−1^, which suggested that SENDs alleviated the INS‐1 cell death induced by stimulation with H_2_O_2_ or PA. The antioxidant effects of SENDs were analyzed further in T2DM mice. The ROS level in T2DM was 6.8‐times higher than that in the healthy mice according to fluorescent staining of tissue (using dihydroethidium as the ROS probe) (Figure 5F,G), which indicated that the death of islet *β* cells was closely related to high levels of ROS. ROS levels in the pancreas of T2DM mice almost returned to normal after SENDs treatment. Meanwhile, the levels of malondialdehyde (MDA) and 8‐Hydroxyguanosine (8‐OHdG) in the pancreas were measured to evaluate the indirect ameliorative effect of SENDs on cellular oxidative damage. Polyunsaturated fatty acids are oxidized readily to generate epoxy‐, oxo‐, or cyclic peroxides by ROS, and MDA is an important end‐product of lipid peroxidation.^[^ ^30^ ^]^ 8‐OHdG is an oxidative adduct produced by ROS attacking the eighth carbon atom of the guanine base in DNA, and is commonly adopted as a biomarker of oxidative damage.^[^ ^31^ ^]^ As shown in Figure 5H–J, levels of MDA and 8‐OHdG were increased significantly in T2DM and restored after SENDs treatment. Especially for 8‐OHdG, SENDs (10 mg kg^−1^) could reduce the 8‐OHdG level to 13.5% of that in the untreated group. Interestingly, in addition to inhibiting gluconeogenesis and improving peripheral insulin resistance, metformin may exert antioxidant effects through multiple pathways such as reducing inflammatory factor expression, upregulating antioxidant enzymes or downregulating NADPH oxidase,^[^ ^24^ ^]^ which is consistent with our experimental results (Figure 5F–J). More importantly, the antioxidant effect of SENDs in low concentrations was certainly superior to that of metformin, which required high daily doses for its therapeutic effect.

GPX1 is the major selenoprotein antioxidant enzyme in islet *β* cells with Sec residues at its active site.^[^ ^32^ ^]^ Direct scavenging of ROS by GPX1 may lead to hyperacetylation of H3 and H4 histones at the proximal promoter of pancreas/duodenum homeobox protein 1 (PDX1), which, in turn, allows an increase in mRNA expression of PDX1 and promotes *β*‐cell maturation and insulin synthesis.^[^ ^33^ ^]^ The high levels of pancreatic Se in T2DM mice were stable over time (Figure 2G), and Se of SENDs should be converted to the GPX1 component to exert a sustained antioxidant effect, probably due to the release of soluble Se ions from SEND after its C‐Se bond was etched by high level of ROS in T2DM islets (Figure 5F). To verify this hypothesis, GPX1 activity and mRNA expression were examined carefully in T2DM mice. As shown in Figure 5D,E, the activity of GPX1 in T2DM mice decreased from 164.9 to 66.5 U L^−1^, and the relative mRNA expression decreased to 50% of that in healthy mice. SENDs enhanced the GPX1 activity greatly to 174.6 U L^−1^, which was twice as high after metformin treatment (85.6 U L^−1^), and increased the mRNA expression (180%), which exceeded the result of metformin treatment (113%). These results suggested that SENDs not only directly served as ROS scavengers, but more importantly, were prodrugs of GPX1 to greatly enhance the weakened antioxidant capacity of *β* cells, maintained the ability of *β* cells to secrete insulin, and allowed them to survive in T2DM.

### 2.5 SENDs Improve Mitophagy

**Figure 6:**
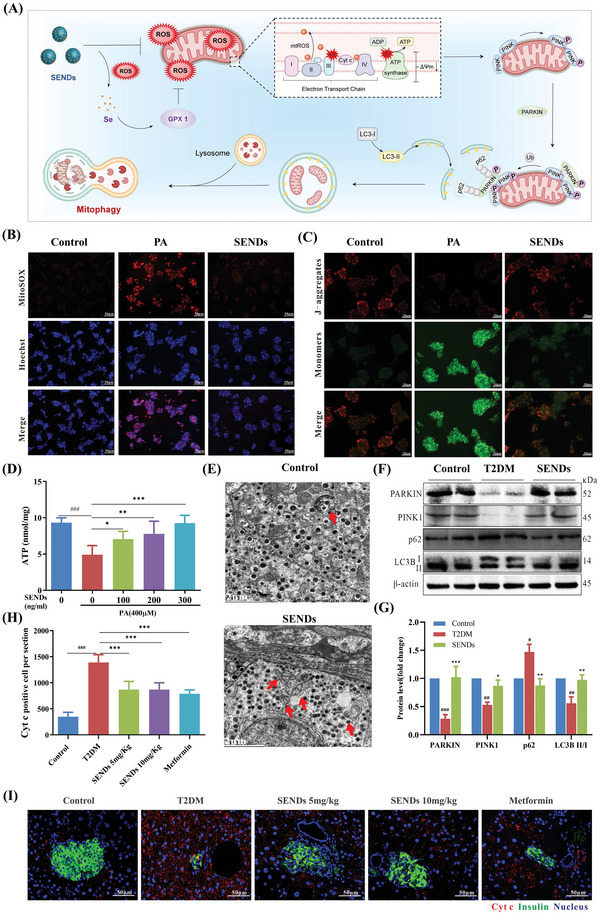
SENDs improve mitophagy. A) Schematic illustration of SENDs improving mitophagy. B) Immunofluorescent staining of MitoSOX (red), Hoechst (blue), and their merge images in INS‐1 cells from each group. Scale bar: 50 µm. C) Immunofluorescent staining of JC‐1 in INS‐1 cells from each group. Scale bar: 50 µm. D) ATP concentrations in palmitic acid‐stimulated INS‐1 cells treated with different concentrations of SENDs. E) TEM images showing mitophagy (red arrow) in islet *β* cells from each group. Scale bar: 2 µm. F,G) Western blot analysis F) and quantification G) of PARKIN, PINK1, p62, and LC3B II/I proteins expression in pancreatic tissues under different treatment conditions. H,I) Cyt c staining (red), insulin (green) staining, and DAPI (blue) staining merge images I) and quantification of Cyt c‐positive cells H) of pancreatic tissues from each group. Scale bar: 50 µm. Data represent means ± S.D. from at least three independent replicates. (^#^ *p* < 0.05, ^##^ *p* < 0.01, ^###^ *p* < 0.001 vs Control group; *p < 0.05, **p < 0.01, ***p < 0.001 vs model group).

Mitochondria, as the “energy factories” of *β* cells, are crucial to the survival and insulin secretion of *β* cells. In T2DM, many toxic metabolites (e.g., fatty acids) interfere with the mitochondrial electron transport chain and induce a large amount of ROS.^[^ ^34^ ^]^ As a result, mitochondria are damaged readily by ROS and then release apoptosis factors (e.g., cytochrome c, Cyt c) to induce *β* cell death.^[^ ^35^ ^]^ Mitophagy degrades dysfunctional mitochondria selectively before damaged mitochondria activate cell death. Therefore, mitophagy can determine the survival or death of *β* cells.^[^ ^36^ ^]^ The classical mitophagy pathway is mediated by PARKIN (an E3 ubiquitin ligase) and PTEN‐induced kinase 1 (PINK1; ubiquitin kinase). If the mitochondrial membrane potential (MMP) is impaired, PINK1 is blocked from entering the inner mitochondrial membrane and accumulates in the outer mitochondrial membrane (OMM), and then recruits PARKIN to ubiquitinate other OMM proteins to form polyubiquitin chains that provide the “eat‐me” signal for autophagy. The bridging protein p62 recognizes this signal and initiates autophagosome formation by binding to microtubule‐associated protein 1 light chain 3 (LC3)‐II. Ultimately, the damaged mitochondria are encapsulated specifically into autophagosomes and fused with lysosomes, thus completing the autophagic degradation of mitochondria and maintaining the stability of the intracellular environment.^[^ ^37^ ^]^ However, mitophagy is inhibited by excessive ROS, and the intracellular accumulation of functionally impaired mitochondria further aggravated mitochondrial and cellular damage.^[^ ^34^ ^]^ Fortunately, SENDs could scavenge mtROS directly or restore the activity of GPX1 (Figure 5D) indirectly to improve mitophagy (**Figure** **6**A). As shown in Figure 6B, SENDs could reduce the production of intracellular PA‐induced mtROS significantly according to a red mitochondrial ROS indicator (MitoSOX). ROS production induced by impaired mitochondria can regulate autophagy in different ways according to ROS level in cell environment. Appropriate ROS promote mitophagy activation to maintain mitochondrial homeostasis, while excessive ROS inhibit mitophagy initiation and lysosomal degradation.^[^ ^38^ ^]^ Based on the significant ROS scavenging efficacy, we further investigated the effect of SENDs on mitophagy. To be noted, SENDs could increase the expression of GPX1 (Figure 5E) and GPX1 is mainly present in the mitochondrial matrix and intermembrane space.^[^ ^39^ ^]^ Therefore, SENDs could eliminate excessive levels of mtROS to restore mitophagy. The ratio of LC3‐II to LC3‐I reflects the number of autophagosomes formed. p62 expression is inversely correlated with LC3‐II expression in normal autophagic flow, and is commonly adopted to detect changes in autophagy.^[^ ^40^ ^]^ As shown in Figure S15 (Supporting Information), PA reduced expression of PARKIN, PINK1, and LC3‐II/I, and increased expression of p62, whereas SENDs reversed this process effectively in INS‐1 cells, indicating that SENDs improved the mitophagy of INS‐1 cells inhibited by excessive ROS. Encouraged by the results in islet *β* cells, the effect of SENDs on mitophagy was explored further in vivo. As shown in Figure 6E, mitophagy was increased significantly through TEM of T2DM islets tissue after SENDs treatment. Moreover, protein expression levels of PARKIN, PINK1, p62, and LC3‐II/I in T2DM mice was 28.5%, 53.1%, 147.1%, and 55.9%, respectively, of that in healthy mice, and returned to normal levels after SENDs treatment, indicating that SENDs effectively ameliorated mitophagy defects under excessive oxidative stress by the PINK/PARKIN pathway (Figure 6F,G). In addition, SENDs reduced Cyt c content in T2DM islets significantly (by 40%), which implied the restoration of mitophagy by SENDs (Figure 6H,I).

Given that the MMP is crucial for maintaining mitochondrial function, MMP was further detected by JC‐1 staining. JC‐1 aggregated in the mitochondrial matrix and generated red fluorescence at a high MMP, whereas JC‐1 retained its monomeric form and generated green fluorescence at a low MMP.^[^ ^41^ ^]^ As shown in Figure 6C, SENDs restored the PA‐induced decrease in the MMP in INS‐1 cells effectively. Finally, the effect of SENDs on ATP content was examined in PA‐damaged mitochondria. As the SENDs concentration increased from 100 to 300 ng mL^−1^, the ATP level increased from 4.9 nmol mg^−1^ in the impaired condition to 7.1 and 9.3 nmol mg^−1^, respectively, which were the same as the ATP content in healthy mice, which further demonstrated the restorative effect of SENDs upon mitochondrial function (Figure 6D).

### 2.6 SENDs Improve ERS and *β* Cell Survival

**Figure 7:**
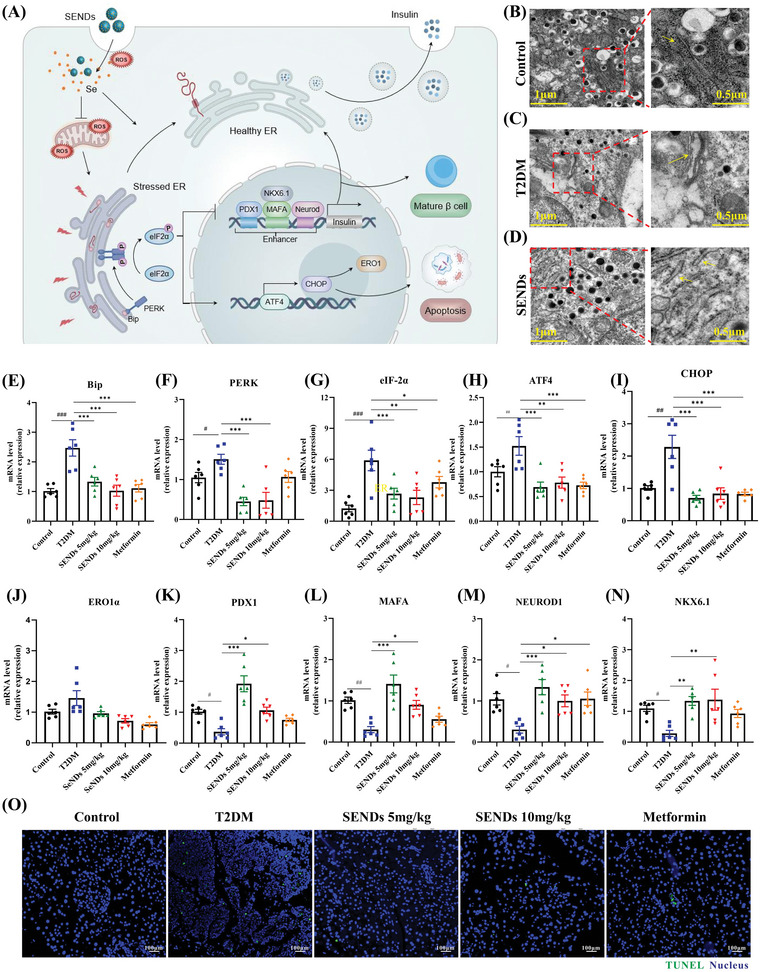
SENDs improve ERS and *β* cell survival. A) Schematic illustration of SENDs to improve endoplasmic reticulum stress and *β*‐cell survival. B–D) TEM images of ER in (B) healthy mice, C) diabetic mice, D) diabetic mice after treatment of SENDs and the magnified images. ER is inside the rectangle on the left and pointed by yellow arrow on the right. E–N) mRNA levels of Bip, PERK, eIF‐2*α*, ATF4, CHOP, ERO1*α*, PDX1, MAFA, NEUROD1, and NKX6.1 in pancreatic tissues homogenates from each group. O) TUNEL staining (green) and DAPI (blue) merge images of pancreatic tissues from each group. Scale bar: 100 µm. Data represent means ± S.D. from at least three independent replicates (^#^ *p* < 0.05, ^##^ *p* < 0.01, ^###^ *p* < 0.001 vs Control group; *p < 0.05, **p < 0.01, ***p < 0.001 vs T2DM group).

Hyperglycemia and oxidative stress lead to *β* cell dysfunction by inducing ERS through the activation of the apoptotic unfolded protein response (UPR) pathways. The ERS is closely associated with T2DM development because the endoplasmic reticulum (ER) is the main site of insulinogen folding, and Ca^2+^ in the ER contributes to insulin secretory signaling.^[^ ^42^ ^]^ GPX1 alleviates ERS directly through the PERK (protein kinase R‐like ER kinase)/eIF‐2*α (α‐subunit of eukaryotic initiation factor 2)*/ATF4 (activating transcription factor 4) pathway, whereas GPX1 deficiency leads to ERS.^[^ ^43^ ^]^ Specifically, when GPX1 is deficient, the conformation of the molecular chaperone immunoglobulin heavy chain binding protein in pre‐B cells (Bip) changes to release the ERS receptor PERK in the ER lumen. Subsequently, the ligand‐independent dimerization domain of PERK is activated to phosphorylate eIF‐2*α* at Ser51 specifically and facilitate the translation ofATF4 to initiate the transcription of C/BEP homologous protein (CHOP), which regulates autophagy‐related genes to promote ERS, apoptosis and upregulated ER oxidoreductin‐1 (ERO1) in islet *β* cells. ERO1,^[^ ^44^ ^]^ as a redox thiolase, plays a crucial role in reoxidizing protein disulfide isomerase and restoring its catalytic capacity for further S–S insertion, and its homeostatic imbalance exacerbates ROS levels and UPR in the ER.^[^ ^45^ ^]^ What's worse, high levels of ROS and severe ERS damage inhibited many critical transcription factors for the development and maturation of islet *β* cells, such as PDX1, V‐maf musculoaponeurotic fibrosarcoma oncogene homolog A (MAFA), neurogenic differentiation 1 (NEUROD1), and NK6 homeobox protein 1 (NKX6.1), which is consistent with the pathological manifestation of *β*‐cell dedifferentiation in T2DM^[^ ^46^ ^]^ (**Figure** **7**A). To this end, ER morphology was first observed using TEM of islet tissues from different groups. The ER structure consisted of a polygonal network of interconnected tubules and lamellae in health islet tissues (Figure 7B). As shown in Figure 7C, the ER in the T2DM group was significantly inflated, damaged, and remained a vague structure. Importantly, the ER structure was restored by SENDs and became only slightly dilated compared with that in the healthy group (Figure 7D). Moreover, the transcription of PERK, eIF‐2*α*, ATF4, CHOP, and other ERS‐related mRNAs was repressed significantly after SENDs increased GPX1 expression, further demonstrating that GPX1 could directly alleviate ERS. As shown in Figure 7E–I, mRNAs expression of Bip, PERK, eIF‐2*α*, ATF4, and CHOP were all increased in the islet tissues of T2DM mice, and SENDs greatly reduced expression of these genes by 46.1%, 14.7%, 54.7%, 54.6%, 43.4% (5 mg kg^−1^). Notedly, ERO1*β* showed the opposite trend while the trend in ERO1*α* isoform changes was the same as that of CHOP in terms of mRNA expression (Figure 7J; and Figure S16, Supporting Information). One possible explanation was that ERO1*β* was more active and sensitive because the *β*‐isoform lacked regulatory cysteines. Therefore, it no longer responded to the upregulated effect of CHOP, and decreased its expression under a too strong oxidative stress stimulus.^[^ ^47^ ^]^ In addition, there was no significant difference in mRNA expression of spliced (s) or total (t) X‐box binding protein 1 (sXBP1 or tXBP1) and inositol‐requiring enzyme‐1*α* (IRE‐1*α*) despite the different ATF6 expression in different groups, excluding the effect of other ERS pathways (Figures S17–S20, Supporting Information). More importantly, mRNA expression of PDX1, MAFA, NEUROD1, and NKX6.1 was also increased and even reversed to above healthy levels after SENDs treatment, whereas the reverse effect could not be achieved with metformin (Figure 7K–N), implying that SENDs could promote the maturation and restore the function of dedifferentiated *β* cells by reversing the metabolic abnormalities. As mentioned above, high levels of ROS induced the release of mitochondrial Cyt c into the cell matrix, while ERS promotes CHOP expression. The mitochondrial and ER pathways leaded to apoptosis synergistically, resulting in the diminished function and number of islet *β* cells. Therefore, apoptosis in different islet tissues were revealed by deoxyribonucleotide end‐transferase (TdT)‐mediated nick end‐labeling (TUNEL) staining. As shown in Figure 7O; and Figure S21 (Supporting Information), the number of apoptotic cells in the T2DM group was about 21.4‐times higher than that in the healthy group, but decreased by nearly 78.5% (5 mg kg^−1^) and 88.8% (10 mg kg^−1^) after SENDs treatment. These data demonstrated that SENDs were extremely efficient in eliminating ERS thus maintaining their numerical and functional stability of *β* cells in T2DM.

### 2.7 Biocompatibility of SENDs

**Figure 8:**
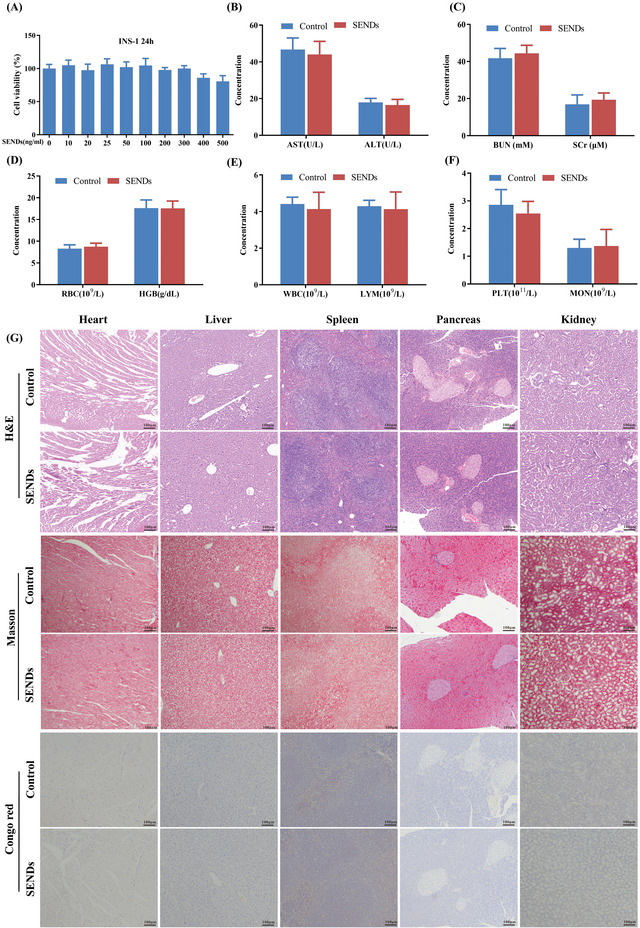
Biocompatibility of SENDs. A) In vitro cytotoxicity of SENDs toward INS‐1 cells after incubation for 24 h. B–F) Serum levels of B) liver function indicators, C) kidney function indicators, and D–F) blood parameters in normal mice (control group) and mice administered intravenously with SENDs, 24 h after injection. G) H&E staining, Masson staining and Congo red staining of major organs (heart, liver, spleen, pancreas, kidney) of normal mice after injection with SENDs (10 mg kg^−1^) for 30 days. Scale bar: 100 µm. Data represent means ± S.D. from six independent replicates.

Finally, the biocompatibility of SENDs was evaluated for future clinical translation from the laboratory to the clinic. The viability of INS‐1 cells was not inhibited even at relatively high concentrations of SENDs according to the cell counting kit‐8 assay, with only a 19% decrease at a concentration of 500 ng mL^−1^ (far higher than the applied concentration) (**Figure** **8**A). Moreover, liver function, kidney function and blood index parameters [erythrocytes (RBC), hemoglobin (HGB), leukocytes (WBC), lymphocytes (LYM), platelets (PLT), monocytes (MON)] were analyzed with whole blood samples of mice. High doses of SENDs (10 mg kg^−1^) did not affect liver function according to levels of aspartate aminotransferase (AST) and alanine aminotransferase (ALT) (Figure 8B). Moreover, SENDs had no adverse effects on the kidney function of mice by the concentration of serum creatine (Scr) and blood urea nitrogen (BUN) (Figure 8C). In addition, SENDs did not affect the blood cell content (Figure 8D–F). Finally, the morphology and structures of the heart, liver, spleen, kidneys, and pancreas of mice were intact after SENDs treatment, and histological abnormalities (cellular wrinkling, fibrosis, and amyloidosis) were not observed (Figure 8G), which were consistent with our previously demonstrated results on SENDs distribution in vivo. SENDs could barely accumulate in sites other than the T2DM‐damaged pancreas, and therefore did not damage normal tissues or organs. These evidences suggested that SENDs had excellent biocompatibility and demonstrated their potential for clinical use.

## 3 Discussion

T2DM pathogenesis is mainly related to the reduction of islet *β*‐cell number and function, insulin resistance, and high levels of ROS within *β* cells. SENDs have two significant advantages over existing Se‐containing nanoparticles: First, compared to many current Se‐containing nanoparticles,^[^ ^48^ ^]^ SENDs have a graphene‐like lamellar structure and smaller size, which ensure that SENDs can be excreted through the kidneys and greatly avoid the damage caused by their accumulation in the body. Common Se or Se‐containing nanoparticles can easily accumulate in the body and cause side effects if injected intravenously, and most of them are administered orally, which greatly limits their bioavailability because of the physical barrier effect of the intestine on nanoparticles. Second, the Se of SENDs is embedded in the conjugated carbon ring through the C—Se bond, and is highly stable under physiological conditions. The release of Se from SENDs only can be induced by high ROS levels in *β* cells after islet‐specific enrichment in T2DM, thus greatly increasing the therapeutic targeting and reducing toxic side effects. Common Se‐containing nanoparticles, which contain generally zero‐valent Se, are unstable under physiological conditions and can be directly oxidized by O_2_ to release high level of soluble Se leading to strong toxic side effects.^[^ ^49^ ^]^

SENDs could effectively restore mitophagy and alleviate ERS in diabetic *β* cells. Large doses of metformin had certain antioxidant effects and similar effects, but they were inferior to SENDs in terms of improving glucose tolerance and increasing insulin levels in T2DM mice. In addition to restoring mitophagy and relieving ERS, SENDs could up‐regulate GPX1 to stimulate important transcription factor expression in *β* cells. To secrete insulin smoothly, *β* cells need to go through multiple developmental stages such as endothelial pancreatic cells, endocrine progenitor cells, immature endocrine cells, mature endocrine cells, and mature *β* cells. PDX1 is necessary for early embryonic development of the pancreas, MAFA participates in activating insulin gene expression,^[^ ^50^ ^]^ NKX6.1 regulates gene expression encoding various proteins responsible for *β* cell proliferation, development and function, glucose uptake, and metabolism as well as insulin biosynthesis are all crucial for *β* cell development and maturation.^[^ ^51^ ^]^ Importantly, PDX1, MAFA, and NKX6.1 all could be restored through elevating GPX1 expression,^[^ ^52^ ^]^ which provided solid evidence for the increase in insulin mRNA and protein expression levels after SENDs treatment.

It is now well established that oxidants have various effects on regulating insulin signaling, and T2DM is being considered as a “redox disease.” Some targets and regulators of insulin receptor signaling are redox sensitive, such as Protein Kinase B (PKB, AKT), Forkhead box O (FOXO), Phosphatase and tensin homolog (PTEN), protein‐tyrosine phosphatase 1B (PTP1B), and JUN amino‐terminal kinase (JNK).^[^ ^9^ ^]^ Oxidation is also required for protein folding in the ER, and hence insufficient supply of oxidants will lead to the UPR activation, which compromises insulin sensitivity in muscle and liver.^[^ ^9^ ^]^ However, excess ROS impairs insulin sensitivity and causes insulin resistance. Binding of insulin to its receptor results in their autophosphorylation of insulin receptor substrates‐1/2 (IRS‐1/2), which activates phosphoinositide 3‐kinase (PI3K), P3 dependent kinase‐1 (PDK‐1), and AKT to guide the translocation of Glucose transporter type 4 (GLUT4) transporter from glucose transport vesicles to cell membrane for glucose uptake and metabolism. Abnormalities in any link in this pathway, leading to impaired insulin receptor signaling, can lead to insulin resistance.^[^ ^53^ ^]^ While high levels of ROS can inhibit the activation of AKT directly or activate JNK to inhibit IRS1/2 autophosphorylation indirectly to cause insulin resistance.^[^ ^53^, ^54^ ^]^ Overall, physiological ROS levels can effectively promote insulin secretion of *β* cells and insulin sensitivity of some key tissues, while insulin resistance and *β* cell apoptosis are closely related to excessive ROS. In our work, SENDs were only highly specifically targeted to the pancreas, with limited distribution in the liver and so on. Therefore. SENDs can be effective in restoring insulin secretion by reducing *β* cell death and restoring *β* cell function thanks to its highly potent antioxidant properties, while SENDs may have limited efficacy in alleviating insulin resistance.

## 4 Conclusions

In recent few decades, more than one‐dozen drugs have been developed vigorously for T2DM treatment after in‐depth exploration of the pathology of T2DM. Such research has greatly improved the quality and intensity of T2DM care and self‐care, and improved quality of life and delayed T2DM complications. However, these drugs are limited effective in preventing islet *β* cells damage, which is the most essential driver of T2DM pathology. Therefore, there is an urgent and unmet need to develop a drug that can effectively rescue the islet *β* cells with long‐term high ROS levels in T2DM.

In this study, we developed a prodrug of Se‐containing SENDs for the repair of damaged islet *β* cells in T2DM. In SEND, Se is embedded into a C‐conjugated backbone through the C—Se covalent bond, which endows SEND with high stability to prevent toxic side‐effects under normal physiological conditions, and achieves specific release of Se as the key GPX1 active factor in islet *β* cells. SENDs can be enriched in islet *β* cells specifically in vivo, greatly increase GPX1 expression by “sending” Se to *β* cells, restore the antioxidant capacity of *β* cells, improve insulin secretion effectively, promote blood sugar back to a normal range and, finally, prevent ROS‐mediated *β* cell death. The therapeutic mechanism of SENDs has also been deeply revealed: SENDs reduce the death of islet *β* cells significantly by alleviating oxidative stress, restoring *β* cell mitophagy, and improving ERS .

In summary, this strategy based on nanomedicine‐based prodrugs can fundamentally solve the defects of traditional antioxidant therapy and achieve long‐term and stable reduction of high levels of ROS in *β* cells. The facile fabrication, economical mass production, and excellent biocompatibility of SENDs indicate their great potential as a novel antioxidant therapy targeting *β* cells in T2DM.

## 5 Experimental Section

### Materials

Stereo fluorescence microscope (M205FCA) were purchased from Leica (German), Mouse insulin (Insulin‐E‐EL‐M1382c) ELISA kits were purchased from Elabscience Biotechnology (Houston, TX). A TUNEL assay kit (C10617), Anti‐fluorescence quenching sealing liquid with DAPI (P36981), Goat anti‐Rabbit IgG (H + L) Highly Cross‐Adsorbed secondary antibody Alexa Fluor 488 (A11029), Goat anti‐Rabbit IgG (H + L) Cross‐Adsorbed secondary antibody Alexa Fluor 555 (A21428), and MitoSOX Red Mitochondrial Superoxide Indicator (M36008) were provided from Thermo Fisher Scientific (Carlsbad, CA). Antibodies against insulin (66198‐1‐lg), glucagon (15954‐1‐AP), and p62 (66184‐1‐lg) were obtained from Wuhan Sanying Biotechnology Co., Ltd (Proteintech Group, Inc). RNAiso Plus(9108/9109), PrimeScript RT reagent Kits with gDNA Eraser(RR047), and RT‐qPCR(RR420A) were bought from Takara Bio Inc (Japan). PARKIN, PINK1, LC3B (D11) XP Rabbit mAb(3836S) were obtained from Cell Signaling Technology. Enhanced mitochondrial membrane potential assay kit with JC‐1(C2003S), Hoechst 33 342 Staining Solution for Live Cells (C1028), and 8‐OHdG (ab48508) were purchased from Beyotime Biotechnology (Shanghai, China). Cyt c antibody (d10933‐1‐AP) was purchased from Proteintech (Rosemont, IL).

### Synthesis of SENDs and FITC‐SENDs

100 mg of L‐selenocysteine was added to 70 mL of ultrapure water, and then NaOH was added to adjust the pH to 9 to dissolve L‐selenocysteine, followed by stirring at 60 °C for 24 h. Subsequently, the precipitate was removed by centrifugation at 12 000 r for 10 min, and the supernatant was dialyzed for 24 h (with four water changes) to remove the unreacted impurities. Finally, the SENDs were obtained by lyophilization.

10 mg of SENDs and 12.5 mg of polyethylene glycol were added to 10 mL of Tris buffer solution and reacted for 12 h at room temperature. After that, the precipitate was dissolved in 8 mL of ultrapure water and then 2 mg of FITC (2 mL in DMSO) was added for 5 h at room temperature in the dark. Subsequently, unreacted FITC was removed by dialysis for 24 h (with four water changes). Finally, the FITC‐SENDs were obtained by lyophilization.

### Characterization of SENDs

TEM images were taken by using a TECNAI G2 high‐resolution transmission electron microscope. XPS measurements (VG ESCALAB MKII) were used to analyze the elemental valence states of SENDs. FTIR was recorded on a Bruker Vertex 70 spectrometer (2 cm^−1^). UV –vis spectra were collected using a VARIAN CARY 50 UV/Vis spectrophotometer. The fluorescence spectra were determined by using an F98 spectrofluorometer. The XRD measurements were carried out using a Bruker D8 Discover X‐ray Diffraction System.

### ROS Scavenging Capacity of SENDs

The O_2_^−·^ scavenging ability of SENDs was detected by the nitro‐blue tetrazolium (NBT) method. In the presence of methionine, riboflavin can be reduced by light, and the reduced riboflavin can be easily reoxidized to produce O_2_^−·^ under aerobic conditions, which can reduce yellow NBT to blue methylhydrazone, which has maximum absorption at 560 nm. In short, Different concentrations of SENDs (15, 30, 60, 120, 240 µg mL^−1^) were mixed with methionine (50 µm), riboflavin (2 µm), NBT (1 mm), PBS (10 mm, pH 7.4), and deionized water. Afterward, the mixture was exposed to UV light for 5 min. Eventually, the O_2_^−·^ scavenging ability of SENDs can be analyzed by comparing the fluorescence intensity.

The ·OH scavenging efficiency of SENDs was determined by fluorescence spectrophotometry. The Fenton reaction between FeSO_4_ and H_2_O_2_ catalyzes the formation of ·OH. Then ·OH combines of nonfluorescent terephthalic acid (TA) and turn it into fluorescent 2‐hydroxyl TA. While, the addition of SENDs can reduce the production of the latter and thus reduce the fluorescence intensity. In brief, different concentrations of SENDs (1, 2, 4, 8, 16, 32 µg mL^−1^) were mixed with benzodicarboxylic acid (0.1 mm), ferrous sulfate (0.05 mm), H_2_O_2_ (1 mm), and PBS (10 mm, pH 7.4). After standing for 6 min, the mixture was transferred to cuvettes and the corresponding fluorescence intensity was scanned under the excitation wavelength of 320 nm.

The H_2_O_2_ scavenging activity of SENDs was determined by UV–vis spectrophotometry. In brief, SENDs (60 µg mL^−1^) and H_2_O_2_ with different concentration (0, 1, 2, 4, 8, 16 mm) were mixed, and then incubated in the dark for 12 h. The clearance rate of H_2_O_2_ was determined by detecting the ultraviolet absorption at 425 nm.

### Animal Experimental Protocols

C57 BL/6 mice (male, 8 weeks, 23–25 g) were purchased from Hunan STA Laboratory Animal CO., LTD (SYXK (XIANG) 2020‐0019) (Changsha, China). All animal study protocol has been approved by the Institutional Animal Care and Use Committee (IACUC), Xiangya Hospital, Central South University, China. These animals were fed with a standard diet and water for 7 days in a clean environment at 24 ± 2 °C and 12 h light/dark cycle. Then, 48 mice were fed with high fat diet (HFD, basic forage 63.5%, lard 8.0%, egg yolk powder 10.0%, saccharose18.0%, sodium cholate 0.5%) for 4 weeks. After that, the HFD‐fed mice were received a daily intraperitoneal injection of streptozocin (STZ, sigma, 60 mg kg^−1^) for 5 days. After the final intraperitoneal injection for 72 h, the mice were then fasted for 12 h. Blood was collected from the tail vein and the fasting blood glucose of each group of mice was measured by glucometer. Fasting blood glucose values ≥ 11.1 mm were considered a modeling success.

12 healthy mice were selected as normal control group and 48 T2DM model mice were used and randomly divided into four groups: 1) HFD+STZ model group, 2) HFD+STZ+SENDs (5 mg kg^−1^), 3) HFD+STZ+SENDs (10 mg kg^−1^), 4) HFD+STZ+Metformin (200 mg kg^−1^), 12 mice in each group. Among them, SENDs were administered by tail vein injection at an interval of 3 days, and metformin was administered by gavage once a day for 4 weeks, and the body weight and fasting glucose of the mice were tested weekly. The blood was harvested after anesthesia, and tissues were collected, weighted, and preserved as soon as possible. Some tissues were fixed in 4% formalin at once, and some tissues were immediately iced in liquid nitrogen and stored in −80 °C. The antidiabetic effects were confirmed by three repeated animal experiments, and the presented results are one representative experiment with 6–12 mice in each group.

### IPGTT

The IPGTT of mice was tested 4 weeks after administration. In the IPGTT, 200 µL of glucose solution was injected into abdominal cavity of mice (1 g kg^−1^) after fasting for 12 h. the blood glucose of mice was measured by tail snipping before and 15, 30, 60, 90, and 120 min after glucose loading. The curve was drawn, and the area under the curve was calculated. IPGTT was confirmed by three repeated animal experiments, and the presented results are one representative experiment with 3–6 mice in each group.

### Biodistribution of SENDs

Normal mice and HFD+STZ mice were given saline and FITC‐SENDs, respectively. Waiting for 6 h, heart, liver, spleen, kidneys, pancreas was collected and placed under a stereo fluorescence microscope (Leica, M205FCA) for observation and image acquisition. After that, the pancreas was frozen at −20 °C for 30 min, embedded in OCT cryopreservative, sectioned, washed 3 times with PBS for 3 min per time, sealed with antifluorescence quencher with DAPI (Invitrogen, P36981), and observed under a fluorescence microscope and images were acquired.

### Hematoxylin and Eosin (HE) Staining

After treatment, pancreatic tail area was fixed in 4%polyformaldehyde for 6 h and embedded in paraffin, then was cut it into slices (5 µm). It was dewaxing following baking at 65 °C for 1 h. Next, the slices were stained by hematoxylin and eosin dyes. At last, Sections were observed under a microscope and photographed.

### Immunofluorescence Analysis

Pancreas tissue was cut into 5 µm sections and deparaffinized with xylene for 7 min (3 times). The slides were gradually rehydrated in various concentrations of alcohol and washed with deionized water. After washing with PBS, the slides were treated with a methanolic solution of 3% of H_2_O_2_ for 20 min at room temperature to quench endogenous peroxide activity. To expose the antigen, the pancreatic sections were boiled in the target repair solution (0.01 m sodium citrate, pH 6.0) for 10 min and then washed with PBS. 0.2% of Triton X‐100 was permeabilized at room temperature for 45 min, 2% of BSA‐TBSTT solution was incubated at room temperature for 45 min, and then washing with TBST three times. Cyt c, 8‐OHdG, Insulin, and Glucagon primary antibody dilutions were used to cover the surface of the sections and incubated overnight at 4 °C. The sections were equilibrated to room temperature and incubated with Alexa Fluor‐488‐coupled goat anti‐rabbit antibody wet box at 37 °C for 1 h. The nuclei were stained with DAPI dropwise and incubated for 3 min at room temperature, and sealed with the anti‐fluorescence bursting agent ProLong Glass Antifade Mountant (invitrogen). The tissue sections were then imaged using a Leica Confocal Laser Scanning Microscope (CLSM) SP8 inverted confocal microscope under three separate laser lines (552, 405, and 488 nm) of excitation.

### Assessment of Pancreatic Superoxide Production

After treatment, the pancreas was collected and stored in optimum cutting temperature (OCT) specimen matrix for cryostat sectioning at −20 °C. The frozen pancreas was further sectioned into tissue slices (≈5 µm thickness) using the Cryotome E freezing microtome (Thermo Carlsbad, CA). To assess superoxide production histologically, frozen pancreatic tissue slices were washed with 1× PBS and stained with Dihydroethidium (DHE, 10 µm) for 30 min. Then it was incubated with DAPI solution at room temperature for 10 min. After washing three times with PBS, each slide was further fixed using coverslips and antifluorescence quenching sealer and fluorescent imaging was recorded on Ortho‐Fluorescent Microscopy (Nikon ECLIPSE C1).

### Apoptosis Analysis

Pancreas tissue apoptosis was measure using TUNEL staining according to the manufacture's protocol, and staining was observed under fluorescence microscope.

### Transmission Electron Microscopy (TEM) of Pancreas

Pancreatic tissues were fixed in fresh TEM fixative and then washed 3 times with 0.1 m PBS (pH 7.4) for 15 min each. Tissues were fixed in 1% OsO_4_ in 0.1 m PB (pH 7.4) for 2 h at room temperature, protected from light. After remove OsO_4_, the tissues were rinsed 3 times in 0.1 m PB (pH 7.4) for 15 min each time. After dehydration at room temperature, the tissue undergoes resin penetration embedding and polymerization. The resin blocks were cut to 60–80 nm thin on the ultramicrotome, and the tissues were fished out onto the 150 meshes cuprum grids with formvar film. They were first stained with 2% uranyl acetate in saturated ethanol solution for 8 min away from light, rinsed with 70% ethanol for 3 times and then rinsed with ultrapure water for 3 times. Next, 2.6% Lead citrate avoid CO_2_ staining for 8 min, and then rinsed with ultrapure water for 3 times. After dried by the filer paper, the cuprum grids were put into the grids board and dried overnight at room temperature. Finally, the cuprum grids were observed and taken images under TEM (HITACHI HT7800/HT77000).

### Quantitative Real‐Time‐PCR Analysis

Total RNA was extracted from frozen pancreas tissues using TRIzol and reverse‐transcribed to obtain cDNA. Quantitative real‐time PCR was performed with Applied Biosystems Step One Plus instrument and TB Green Premix Ex TaqTM (Tli RNaseH Plus). Gene expression was evaluated by the comparative‐Ct Method, using *β*‐actin as a reference gene. The sequences of primers are listed in **Table** **1**.

**Table 1:** Primers used for quantitative real‐time PCR

Target gene	Primer	Sequence (5´‐3´)
Insulin	Forward	CTTTTGTCAAGCAGCACCTTT
	Reverse	GGACATGGGTGTGTAGAAGAAG
GPX1	Forward	ACAGTCCACCGTGTATGCCTTC
	Reverse	CTCTTCATTCTTGCCATTCTCCTG
PDX1	Forward	GAGCGTTCCAATACGGACCA
	Reverse	TCAGCCGTTCTGTTTCTGGG
MAFA	Forward	GCTGGTATCCATGTCCGTGC
	Reverse	GTCGGATGACCTCCTCCTTG
NKX6.1	Forward	CTTCTGGCCCGGAGTGATG
	Reverse	GGGTCTGGTGTGTTTTCTCTTC
NEUROD1	Forward	ACGCAGAAGGCAAGGTGTCC
	Reverse	TTGGTCATGTTTCCACTTCC
CHOP	Forward	GTCCCTGCCTTTCACCTTGG
	Reverse	GGTTTTTGATTCTTCCTCTTCG
Bip	Forward	TGTGTGTGAGACCAGAACCG
	Reverse	TAGGTGGTCCCCAAGTCGAT
PERK	Forward	AGGCTTTAACTTCCCGCATT
	Reverse	AGTGCCAGACTGAAAGTAAATACG
eIF‐2*α*	Forward	CCGCTCTTGACAGTCCGAG
	Reverse	GCAGTAGTCCCTTGTTAGTGACA
ATF4	Forward	CCTGAACAGCGAAGTGTTGG
	Reverse	TGGAGAACCCATGAGGTT
IRE‐1*α*	Forward	CCTACAAGAGTATGTGGAGC
	Reverse	GGTCTCTGTGAACAATGTTGAGAG
sXBP1	Forward	CTGAGTCCGAATCAGGTGCAG
	Reverse	GTCCATGGGAAGATGTTCTGG
tXBP1	Forward	CAGCACTCAGACTATGTGCA
	Reverse	GTCCATGGGAAGATGTTCTGG
ATF6	Forward	TCGCCTTTTAGTCCGGTTCTT
	Reverse	GGCTCCATAGGTCTGACTC
*β*‐actin	Forward	ACGAGGCCCAGAGCAAGA
	Reverse	TTGGTTACAATGCCGTGTTCA

### Cell Culture

The INS‐1 cells were cultured in RMPI1640 medium supplemented with 12% fetal bovine serum (FBS), 10 mm HEPES, 50 m 2‐mercaptoethanol, and 2 mm glutamine at 37 °C in an incubator supplied with a humidified atmosphere of 5% CO_2_.

### Cytotoxicity Assay

INS‐1 cells were seeded into 96‐well plates and treated with different concentrations of SENDs after 24 h of treatment with or without PA. Cell viability was tested using CCK‐8 reagent at 10 µL per well for 2 h at 37 °C. Use a microplate reader to detect the optical density (OD) value at 450 nm.

### Western Blotting

Proteins were extracted from pancreatic tissue or cells using RIPA buffer containing PMSF and phosphatase inhibitors. The supernatants of the mixture were obtained by centrifugation at 4 °C, and their total protein concentrations were further determined using the BCA Protein Assay kit. Protein extracts were separated on sodium dodecyl sulfate‐polyacrylamide gel electrophoresis and blotted onto PVDF membranes. After blocking with fat‐free milk, the membranes were incubated at 4 °C overnight with the primary antibody. The bound antibodies were detected using horseradish peroxidase (HRP)‐conjugated IgG and visualized with enhanced chemiluminescence detection reagents. Protein bands were evaluated using Image J software.

### Mitochondrial Function

Live INS‐1 cells after different treatments were labeled with 5 µm MitoSOX reagent at 37 °C for 10 min, then stained with Hoechst 33 342 live cell staining solution and finally placed in warm buffer. Mitochondrial membrane potential was measured using JC‐1 staining working solution at 37 °C for 15 min. After incubation, the cells were washed for several times and examined using fluorescence microscope.

### ATP Assays

INS‐1 cells with different treatments were harvested by centrifugation (14 000 G, 4 °C, 2 min) and washed with precooled PBS. ATP content was determined using the Enhanced ATP Assay Kit. Add the detection solution to the white 96‐well plate and incubate for 5 min at room temperature. Then the supernatant of the lysed cells was quickly mixed with the detection solution, and read after 30 min.

### Biochemical Assays

Pancreas of mice collected from each group were cryopreserved in a refrigerator at −80 °C, and the renal homogenates were obtained for different experiments. Insulin levels in pancreatic tissue homogenates were measured by an enzyme‐linked immunosorbent assay (ELISA) kit according to the manufacturer's protocol. An MDA assay kit was utilized to measure the level of lipid peroxidation in tissues.

### Primary Islet Isolation

The mice were sacrificed and soaked in 75% alcohol for disinfection. Transfer to aseptic conditions, expose the abdominal cavity, and ligate the common bile duct, and retrogradely perfuse 3 mL of 1 mg mL^−1^ Collagen P precooled HBSS Buffer containing DNAase along the common bile duct. Immediately after the pancreas is inflated, the pancreatic tissue is removed. Digest at 37 °C for 10 min, shake manually 5–10 times, and add 50 mL of precooled 10% FBS HBSS Buffer to terminate the digestion. Centrifuge at 300G for 3 min to remove the supernatant. Add precooled 10% FBS HBSS Buffer to resuspend, filter through 500 µm gauze and centrifuge at 300G for 3 min to collect the precipitate. Use 10 mL precooled histopaque 1.119 to resuspend the pellet, and then gently add 10 mL histopaque 1.077 and 10 mL HBSS Buffer. Centrifuge at 2000 rpm with brake off for 30 min, carefully collect the islet layer, and select islets to introduce into fresh medium.

### GSIS

INS‐1 cells in logarithmic phase were seeded in 24‐well plates at a density of 2×10^5^ per well and cultured to 80–90% confluence. The 24‐well plate was washed once with glucose‐free Krebs‐Ringer bicarbonate HEPES (glucose‐free KRBH buffer, pH 7.4, containing :135 mm NaCl, 3.6 mm KCl, 5 mm NaHCO_3_, 0.5 mm NaH_2_PO_4_, 0.5 mm MgCl_2_, 1.5 mm CaCl_2_, 10 mm HEPES, BSA 0.1%) solution, incubated at 37 °C for 1 h, and the supernatant was removed.

Add 2 islets derived from T2DM mice to each well of a 96‐well plate, incubate with glucose‐free KRBH at 37 °C for 30 min, and remove the supernatant. Add 0.5 mL of KRBH solution containing 2.8 and 20 mm glucose and incubate at 37 °C for 1 h, centrifuge (1000 rpm, 5 min) to take the supernatant and save the insulin to be tested, which will be used as the secretion of basal insulin and the secretion of insulin stimulated by high glucose respectively. Insulin concentration in the supernatant was determined using an insulin ELISA kit.

### In Vivo Toxicity Assessment

Healthy C57 BL/6 mice were randomized into two groups. The control group, consisted of healthy mice injected with 100 µL of 1×PBS (*n* = 6); the SENDs group consisted of healthy mice injected with 100 µL of SENDs 10 mg kg^−1^ in 1× PBS (*n* = 6). Healthy mice were sacrificed 24 h or 30 days postinjection. Histological changes in the organs of the mice were assessed to evaluate the acute and long‐term toxicity of the SENDs by H&E staining, Masson staining and Congo red staining. Whole blood was used for hematological analysis with the following parameters: RBC, HGB, WBC, LYM, PLT, MON. Indicators of hepatic function (ALT and AST) and kidney function (BUN and creatinine levels) were analysis using automatic biochemistry analyzer BS‐2000 M.

### Statistical Analysis

All of the data were presented as the mean ± SEM. from a minimum of three independent experiments. Data analysis was performed using SPSS 22.0 (SPSS Inc., USA), Image J (version 1.8.0), and GraphPad Prism Software Version 8.0 (GraphPad Prism, San Diego, USA). For statistical comparison, one‐way ANOVA Tukey's post‐hoc analysis was performed. A value of *p* < 0.05 was considered significant.

## Conflict of Interest

The authors declare no conflict of interest.

## Supporting information

Supporting Information

## Data Availability

The data that support the findings of this study are available from the corresponding author upon reasonable request.

## References

[1] Explore results from the 2019 Global Burden of Disease (GBD) study[EB/OL]. [Z].

[2] S. Bellary , I. Kyrou , J. E. Brown , C. J. Bailey , Nat. Rev. Endocrinol. 2021, 17, 534. 34172940 10.1038/s41574-021-00512-2

[3] D. Tomic , J. E. Shaw , D. J. Magliano , Nat Rev Endocrinol 2022, 18, 525. 35668219 10.1038/s41574-022-00690-7 PMC9169030

[4] S. K. Gunasekar , L. Xie , A. Kumar , J. Hong , P. R. Chheda , C. Kang , D. M. Kern , C. My‐Ta , J. Maurer , J. Heebink , E. E. Gerber , W. J. Grzesik , M. Elliot‐Hudson , Y. Zhang , P. Key , C. A. Kulkarni , J. W. Beals , G. I. Smith , I. Samuel , J. K. Smith , P. Nau , Y. Imai , R. D. Sheldon , E. B. Taylor , D. J. Lerner , A. W. Norris , S. Klein , S. G. Brohawn , R. Kerns , R. Sah , Nat. Commun. 2022, 13, 784. 35145074 10.1038/s41467-022-28435-0 PMC8831520

[5] D. E. James , J. Stöckli , M. J. Birnbaum , Nat. Rev. Mol. Cell Biol. 2021, 22, 751. 34285405 10.1038/s41580-021-00390-6

[6] G. C. Weir , J. Gaglia , S. Bonner‐Weir , Lancet Diabetes Endocrinol. 2020, 8, 249. 32006519 10.1016/S2213-8587(20)30022-X PMC7098467

[7] L. Perreault , J. S. Skyler , J. Rosenstock , Nat. Rev. Endocrinol. 2021, 17, 364. 33948015 10.1038/s41574-021-00489-y

[8] M. Y. Donath , C. A. Dinarello , T. Mandrup‐Poulsen , Nat. Rev. Immunol. 2019, 19, 734. 31501536 10.1038/s41577-019-0213-9

[9] H. Sies , D. P. Jones , Nat. Rev. Mol. Cell Biol. 2020, 21, 363. 32231263 10.1038/s41580-020-0230-3

[10] S. Baumel‐Alterzon , L. S. Katz , G. Brill , A. Garcia‐Ocaña , D. K. Scott , Trends Endocrinol. Metab. 2021, 32, 7. 33243626 10.1016/j.tem.2020.11.002 PMC7746592

[11] V. Poitout , R. P. Robertson , Endocr. Rev. 2008, 29, 351. 18048763 10.1210/er.2007-0023 PMC2528858

[12] W. Ying , W. Fu , Y. S. Lee , J. M. Olefsky , Nat. Rev. Endocrinol. 2020, 16, 81. 31836875 10.1038/s41574-019-0286-3 PMC8315273

[13] J. Ambati , J. Magagnoli , H. Leung , S.‐B. Wang , C. A. Andrews , D. Fu , A. Pandey , S. Sahu , S. Narendran , S. Hirahara , S. Fukuda , J. Sun , L. Pandya , M. Ambati , F. Pereira , A. Varshney , T. Cummings , J. W. Hardin , B. Edun , C. L. Bennett , K. Ambati , B. J. Fowler , N. Kerur , C. Röver , N. Leitinger , B. C. Werner , J. D. Stein , S. S. Sutton , B. D. Gelfand , Nat. Commun. 2020, 11, 4737. 32968070 10.1038/s41467-020-18528-z PMC7511405

[14] H. J. Forman , H. Zhang , Nat. Rev. Drug Discovery 2021, 20, 689. 34194012 10.1038/s41573-021-00233-1 PMC8243062

[15] L. Cottle , W. J. Gan , I. Gilroy , J. S. Samra , A. J. Gill , T. Loudovaris , H. E. Thomas , W. J. Hawthorne , M. A. Kebede , P. Thorn , Diabetologia 2021, 64, 618. 33399909 10.1007/s00125-020-05345-8 PMC7864831

[16] C. Yang , M. Eleftheriadou , S. Kelaini , T. Morrison , M. V. González , R. Caines , N. Edwards , A. Yacoub , K. Edgar , A. Moez , A. Ivetic , A. Zampetaki , L. Zeng , F. L. Wilkinson , N. Lois , A. W. Stitt , D. J. Grieve , A. Margariti , Nat. Commun. 2020, 11, 3812. 32732889 10.1038/s41467-020-17468-y PMC7393072

[17] J. Zhao , H. Zou , Y. Huo , X. Wei , Y. Li , Front. Nutr. 2022, 9, 1027629. 36438755 10.3389/fnut.2022.1027629 PMC9686347

[18] Y. Shi , W. Du , W. Zhou , C. Wang , S. Lu , S. Lu , B. Zhang , Angew. Chem., Int. Ed. Eng. 2020, 59, 22470. 10.1002/anie.202011097 32897620

[19] O. I. Brown , K. I. Bridge , M. T. Kearney , Cells 2021, 10, 2315. 34571964 10.3390/cells10092315 PMC8469180

[20] M. J. Quizon , A. J. García , Annu. Rev. Pathol. 2022, 17, 485. 34813353 10.1146/annurev-pathol-042320-094846

[21] C. G. Schalkwijk , C. D. A. Stehouwer , Physiol. Rev. 2020, 100, 407. 31539311 10.1152/physrev.00001.2019

[22] Q. Chen , Y. Nan , Y. Yang , Z. Xiao , M. Liu , J. Huang , Y. Xiang , X. Long , T. Zhao , X. Wang , Q. Huang , K. Ai , Bioact. Mater. 2023, 22, 141. 36203963 10.1016/j.bioactmat.2022.09.021 PMC9526023

[23] AMERICAN DIABETES A , Diabetes Care 2019, 42, S13. 30559228 10.2337/dc19-S002

[24] N. Apostolova , F. Iannantuoni , A. Gruevska , J. Muntane , M. Rocha , V. M. Victor , Redox Biol. 2020, 34, 101517. 32535544 10.1016/j.redox.2020.101517 PMC7296337

[25] J. E. Campbell , C. B. Newgard , Nat. Rev. Mol. Cell Biol. 2021, 22, 142. 33398164 10.1038/s41580-020-00317-7 PMC8115730

[26] P. Acosta‐Montaño , E. Rodríguez‐Velázquez , E. Ibarra‐López , H. Frayde‐Gómez , J. Mas‐Oliva , B. Delgado‐Coello , I. A. Rivero , M. Alatorre‐Meda , J. Aguilera , L. Guevara‐Olaya , V. García‐González , Cells 2019, 8, 884. 31412623 10.3390/cells8080884 PMC6721695

[27] Q. Ma , Y. Xiao , W. Xu , M. Wang , S. Li , Z. Yang , M. Xu , T. Zhang , Z.‐N. Zhang , R. Hu , Q. Su , F. Yuan , T. Xiao , X. Wang , Q. He , J. Zhao , Z.‐J. Chen , Z. Sheng , M. Chai , H. Wang , W. Shi , Q. Deng , X. Cheng , W. Li , Nat. Commun. 2022, 13, 4142. 35842441 10.1038/s41467-022-31829-9 PMC9288460

[28] P. Llanos , A. Contreras‐Ferrat , G. Barrientos , M. Valencia , D. Mears , C. Hidalgo , PLoS One 2015, 10, e0129238. 26046640 10.1371/journal.pone.0129238 PMC4457734

[29] Q. Huang , Y. Yang , Y. Zhu , Q. Chen , T. Zhao , Z. Xiao , M. Wang , X. Song , Y. Jiang , Y. Yang , J. Zhang , Y. Xiao , Y. Nan , W. Wu , K. Ai , Small 2023, 19, 2207350. 10.1002/smll.202207350 36760016

[30] M. P. Murphy , H. Bayir , V. Belousov , C. J. Chang , K. J. A. Davies , M. J. Davies , T. P. Dick , T. Finkel , H. J. Forman , Y. Janssen‐Heininger , D. Gems , V. E. Kagan , B. Kalyanaraman , N.‐G. Larsson , G. L. Milne , T. Nyström , H. E. Poulsen , R. Radi , H. Van Remmen , P. T. Schumacker , P. J. Thornalley , S. Toyokuni , C. C. Winterbourn , H. Yin , B. Halliwell , Nat. Metab. 2022, 4, 651. 35760871 10.1038/s42255-022-00591-z PMC9711940

[31] J. A. Jamsen , A. Sassa , L. Perera , D. D. Shock , W. A. Beard , S. H. Wilson , Nat. Commun. 2021, 12, 5055. 34417448 10.1038/s41467-021-24486-x PMC8379156

[32] D. Yan , G. Wang , F. Xiong , W.‐Y. Sun , Z. Shi , Y. Lu , S. Li , J. Zhao , Nat. Commun. 2018, 9, 4293. 30327477 10.1038/s41467-018-06763-4 PMC6191425

[33] X. D. Wang , M. Z. Vatamaniuk , S. K. Wang , C. A. Roneker , R. A. Simmons , X. G. Lei , Diabetologia 2008, 51, 1515. 18560803 10.1007/s00125-008-1055-3

[34] S. J. Forrester , D. S. Kikuchi , M. S. Hernandes , Q. Xu , K. K. Griendling , Circ. Res. 2018, 122, 877. 29700084 10.1161/CIRCRESAHA.117.311401 PMC5926825

[35] I. Vercellino , L. A. Sazanov , Nat. Rev. Mol. Cell Biol. 2022, 23, 141. 34621061 10.1038/s41580-021-00415-0

[36] F. J. Bock , S. W. G. Tait , Nat. Rev. Mol. Cell Biol. 2020, 21, 85. 31636403 10.1038/s41580-019-0173-8

[37] K. Palikaras , E. Lionaki , N. Tavernarakis , Nat. Cell Biol. 2018, 20, 1013. 30154567 10.1038/s41556-018-0176-2

[38] H. Sun , T. Ou , J. Hu , Z. Yang , Q. Lei , Y. Li , G. Wang , Y. Li , K. Wu , S. Wang , S. Wu , Biochem. Pharmacol. 2021, 190, 114588. 33957094 10.1016/j.bcp.2021.114588

[39] V. Ribas , C. Garcá­A‐Ruiz , J. C. FernáNdez‐Checa , Front. Pharmacol. 2014, 5, 151. 25024695 10.3389/fphar.2014.00151 PMC4079069

[40] Y. Ding , D. Xing , Y. Fei , B. Lu , Chem. Soc. Rev. 2022, 51, 8832. 36218065 10.1039/d2cs00624c PMC9620493

[41] Q. Huang , Y. Yang , T. Zhao , Q. Chen , M. Liu , S. Ji , Y. Zhu , Y. Yang , J. Zhang , H. Zhao , Y. Nan , K. Ai , Bioact. Mater. 2023, 21, 381. 36185743 10.1016/j.bioactmat.2022.08.022 PMC9483742

[42] D. L. Eizirik , L. Pasquali , M. Cnop , Nat. Rev. Endocrinol. 2020, 16, 349. 32398822 10.1038/s41574-020-0355-7

[43] P. Geraghty , N. Baumlin , M. A. Salathe , R. F. Foronjy , J. M. D'armiento , Mediators Inflamm. 2016, 2016, 9461289. 28070146 10.1155/2016/9461289 PMC5187475

[44] J. Yong , J. D. Johnson , P. Arvan , J. Han , R. J. Kaufman , Nat. Rev. Endocrinol. 2021, 17, 455. 34163039 10.1038/s41574-021-00510-4 PMC8765009

[45] L. Wang , C.‐C. Wang , Trends Biochem. Sci. 2023, 48, 40. 35871147 10.1016/j.tibs.2022.06.011

[46] M. Liu , Y. Huang , X. Xu , X. Li , M. Alam , A. Arunagiri , L. Haataja , L. Ding , S. Wang , P. Itkin‐Ansari , R. J. Kaufman , B. Tsai , L. Qi , P. Arvan , J. Clin. Invest. 2021, 131, e142240. 33463547 10.1172/JCI142240 PMC7810482

[47] A. G. Shergalis , S. Hu , A. Bankhead , N. Neamati , Pharmacol. Ther. 2020, 210, 107525. 32201313 10.1016/j.pharmthera.2020.107525 PMC7316501

[48] A. A.‐R. Mohamed , S. I. Khater , A. Hamed Arisha , M. M. M. Metwally , G. Mostafa‐Hedeab , E. S. El‐Shetry , Gene 2021, 768, 145288. 33181259 10.1016/j.gene.2020.145288

[49] L. Wang , C. Li , Q. Huang , X. Fu , Food Funct. 2019, 10, 539. 30662993 10.1039/c8fo01958d

[50] Y. Zhu , Q. Liu , Z. Zhou , Y. Ikeda , Stem Cell Res. Ther. 2017, 8, 240. 29096722 10.1186/s13287-017-0694-z PMC5667467

[51] I. I. Aigha , E. M. Abdelalim , Stem Cell Res. Ther. 2020, 11, 459. 33121533 10.1186/s13287-020-01977-0 PMC7597038

[52] H. Steinbrenner , L. H. Duntas , M. P. Rayman , Redox Biol. 2022, 50, 102236. 35144052 10.1016/j.redox.2022.102236 PMC8844812

[53] R. G. Kerry , G. P. Mahapatra , G. K. Maurya , S. Patra , S. Mahari , G. Das , J. K. Patra , S. Sahoo , Rev. Endocr. Metab. Disord. 2021, 22, 421. 33052523 10.1007/s11154-020-09606-0

[54] E. J. Anderson , M. E. Lustig , K. E. Boyle , T. L. Woodlief , D. A. Kane , C.‐T. Lin , J. W. Price , L. Kang , P. S. Rabinovitch , H. H. Szeto , J. A. Houmard , R. N. Cortright , D. H. Wasserman , P. D. Neufer , J. Clin. Invest. 2009, 119, 573. 19188683 10.1172/JCI37048 PMC2648700

