# Supplementary material for: Selenium Nanodots (SENDs) as Antioxidants and Antioxidant‐Prodrugs to Rescue Islet β Cells in Type 2 Diabetes Mellitus by Restoring Mitophagy and Alleviating Endoplasmic Reticulum Stress
Source: Adv Sci (Weinh). 2023 Apr 21;10(19):2300880. doi: 10.1002/advs.202300880 (PMC10323656; doi:10.1002/advs.202300880)
Supplement: Supplementary file 1 — Supporting Information [file ADVS-10-2300880-s001.pdf]

ADVANCED SCIENCE
Open Access

Supporting Information

for *Adv. Sci.*, DOI 10.1002/advs.202300880

Selenium Nanodots (SENDs) as Antioxidants and Antioxidant-Prodrugs to Rescue Islet *β* Cells in Type 2 Diabetes Mellitus by Restoring Mitophagy and Alleviating Endoplasmic Reticulum Stress

*Qiong Huang, Zerun Liu, Yunrong Yang, Yuqi Yang, Ting Huang, Ying Hong, Jinping Zhang, Qiaohui Chen, Tianjiao Zhao, Zuoxiu Xiao, Xuejun Gong, Yitian Jiang, Jiang Peng, Yayun Nan and Kelong Ai**

Supporting Information

Selenium nanodots (SENDs) as antioxidants and antioxidant-prodrugs to rescue islet β cells in type 2 diabetes mellitus by restoring mitophagy and alleviating endoplasmic reticulum stress

Qiong Huang[1,2], Zerun Liu[1,2], Yunrong Yang[1,2], Yuqi Yang[1,2], Ting Huang[1,2], Ying Hong[1,2], Jinping Zhang[1,2], Qiaohui Chen[3,4], Tianjiao Zhao[3,4], Zuoxiu Xiao[3,4], Xuejun Gong[5], Yitian Jiang[3,4], Jiang Peng[3,4], Yayun Nan[6], Kelong Ai [3,4]*

[1] Department of Pharmacy, Xiangya Hospital, Central South University, Changsha, 410008, China

[2] National Clinical Research Center for Geriatric Disorders, Xiangya Hospital, Central South University, Changsha, 410008, China

[3] Xiangya School of Pharmaceutical Sciences, Central South University, Changsha, 410078, China

[4] Hunan Provincial Key Laboratory of Cardiovascular Research, Xiangya School of Pharmaceutical Sciences, Central South University, Changsha, 410078, China

[5] Pancreatic Surgery, Xiangya Hospital, Central South University, Changsha, 410008, China

[6] Geriatric Medical Center, People's Hospital of Ningxia Hui Autonomous Region, Yinchuan, 750002, China

***Corresponding authors**: aikelong@csu.edu.cn (Prof. Kelong Ai)

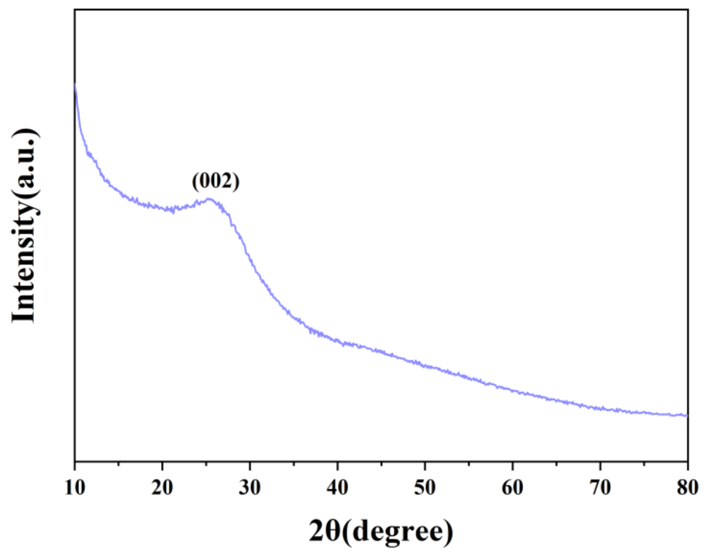

Figure S1 X-ray diffraction (XRD) pattern of SENDs

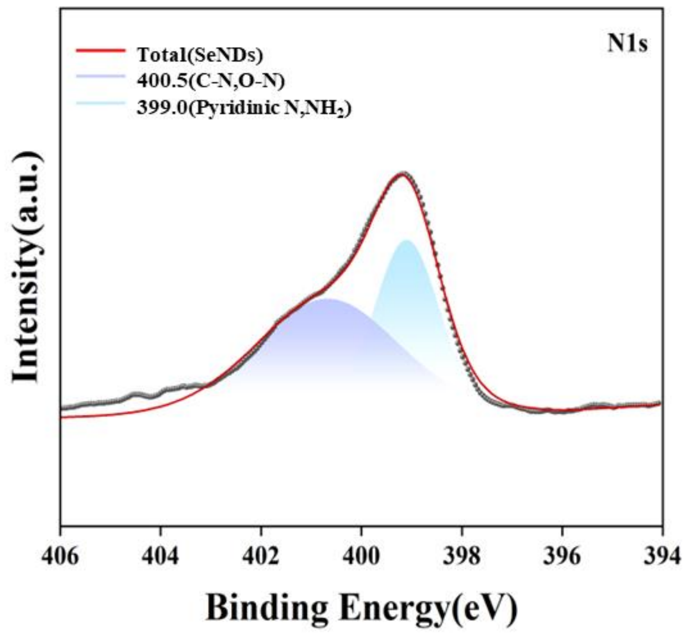

Figure S2 XPS spectrum of N1s in SENDs.

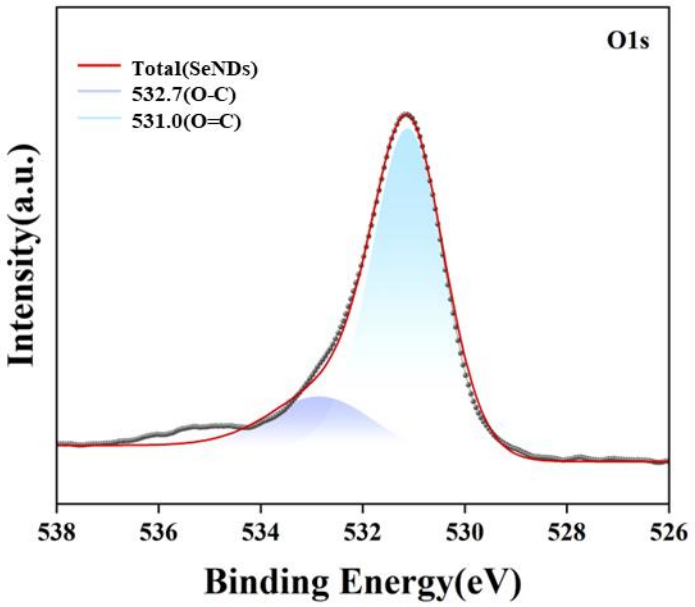

Figure S3 XPS spectrum of O1s in SENDs.

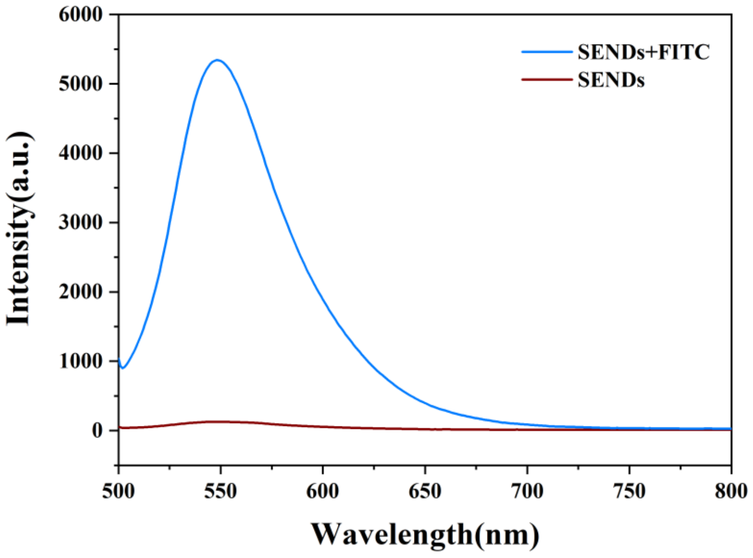

Figure S4 Fluorescence spectrum of SENDs and FITC-SENDs.

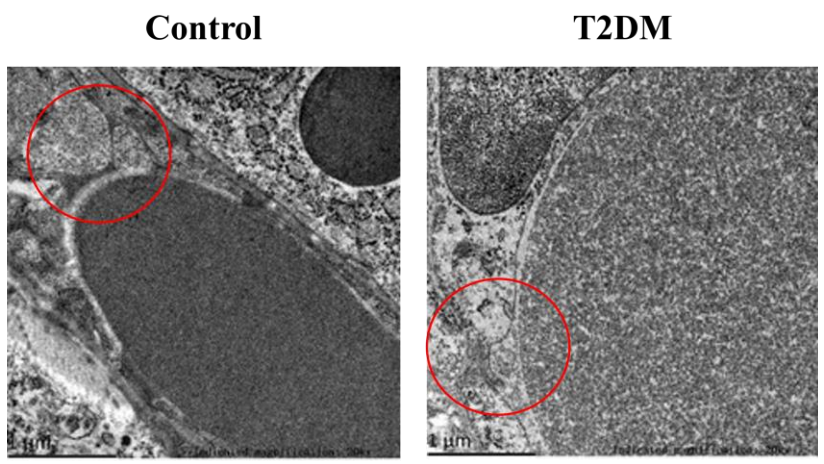

Figure S5 TEM images showing endothelial junction (red cycle) in islets from each group. Scale bar: 1 μm.

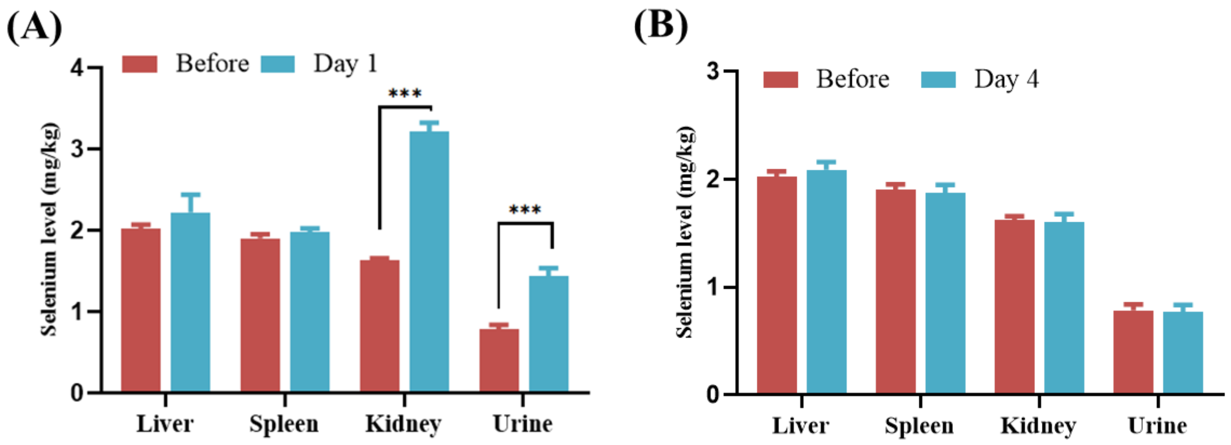

Figure S6 Quantification of the selenium content in organs and urine was determined by ICP at day 1 (A) and day 4 (B). Data represent means ± S.D. from at least three independent replicates. (****P*<0.001).

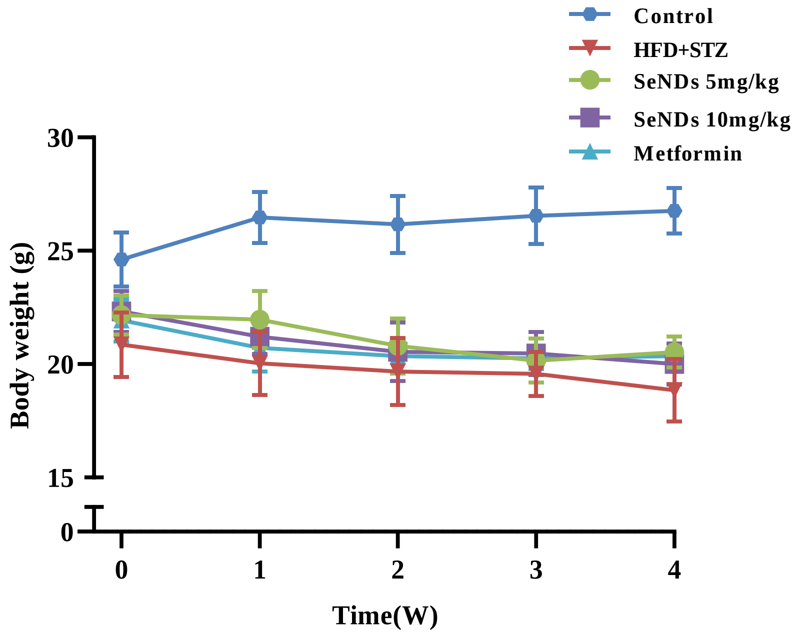

Figure S7 Every week body weight development during the drug treatment from each group. Data represent means ± S.D. from six independent replicates.

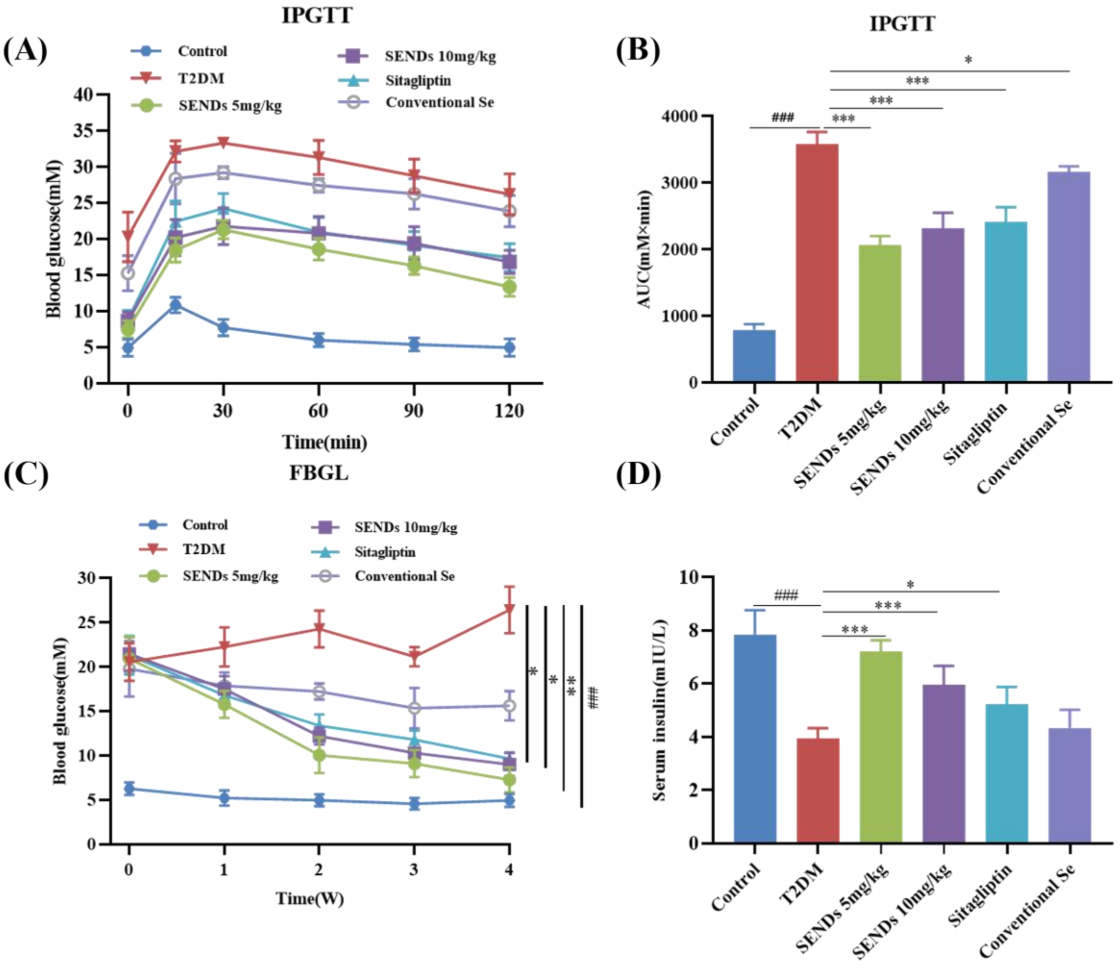

Figure S8 (A-B) IPGTT was measured after three weeks of drug administration (A) and the AUC of IPGTT (B). (C) The FBGL of mice during the drug treatment. (D) The serum insulin level from each group. Data represent means ± S.D. from at least three independent replicates. ($^{###}$*P*<0.001 *vs* Control group, **P*<0.05, ***P*<0.01, ****P*<0.001, *vs* T2DM group).

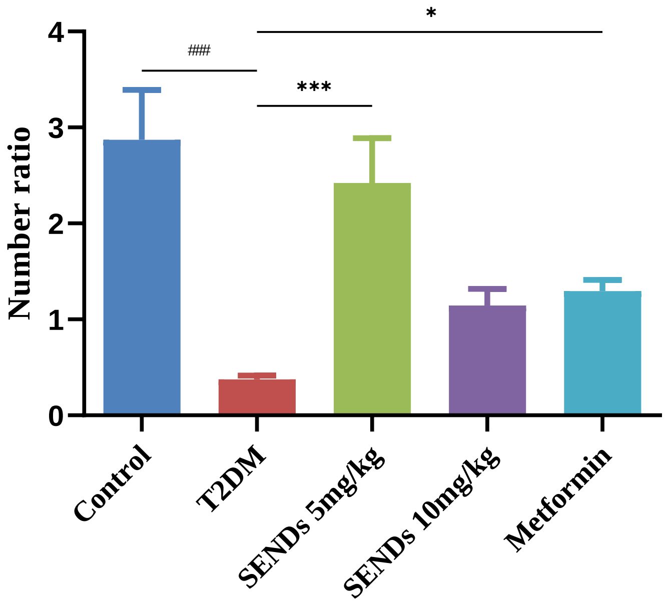

Figure S9 Ratio of the number of β cells to α cells. Data from quantitative analysis of insulin and glucagon immunofluorescence results. Data represent means ± S.D. from at least three independent replicates. ($^{###}$*P*<0.001 vs Control group, **P*<0.05, ****P*<0.001 *vs* T2DM group).

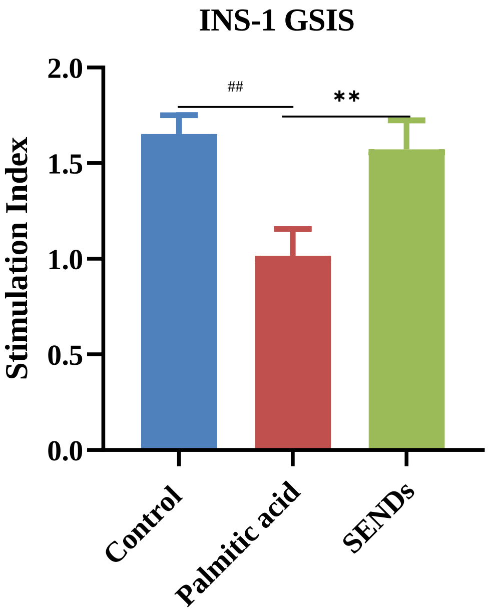

Figure S10 Stimulation index of GSIS experiments of INS-1 cell from each group. Data represent means ±

S.D. from at least three independent replicates. ($^{\#\#}P$<0.01 vs Control group, $^{**}P$<0.01 *vs* Palmitic acid group).

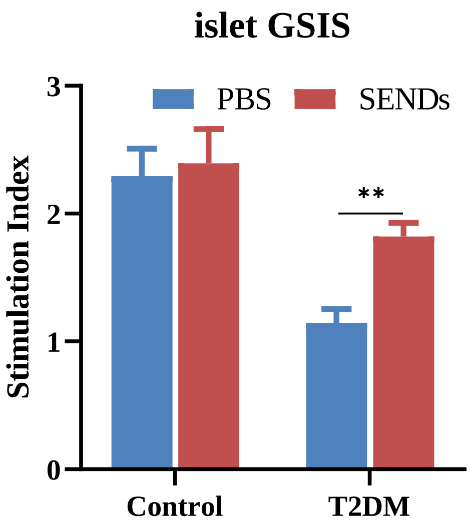

Figure S11 Stimulation index of GSIS experiments of pancreatic islet-like organs from each group. Data represent means ± S.D. from at least three independent replicates. ($^{**}P$<0.01 *vs* PBS group).

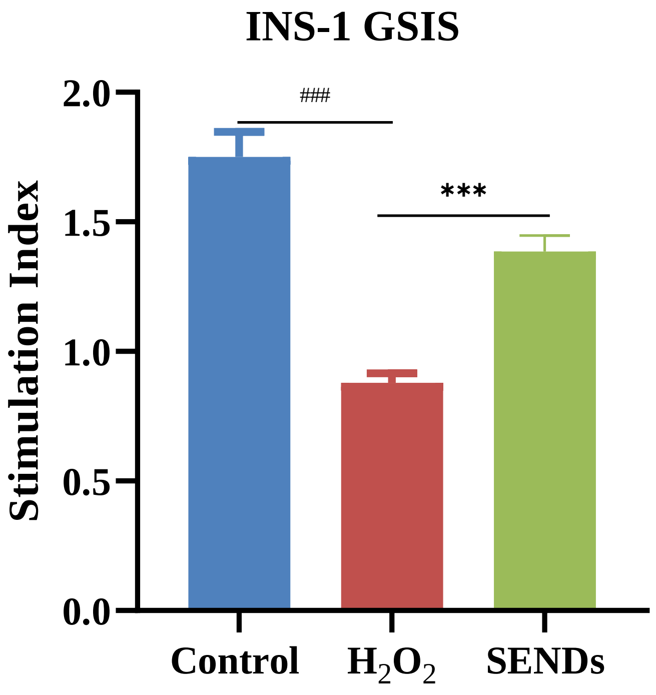

Figure S12 Stimulation index of GSIS experiments of INS-1 cells from each group. Data represent means ± S.D. from at least three independent replicates. ($^{\#\#\#}P$<0.001 *vs* Control group, $^{***}P$<0.001 *vs* H_2O_2 group).

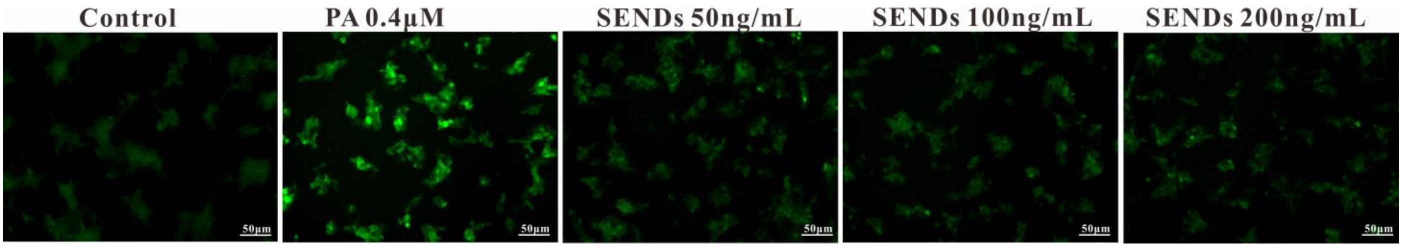

Figure S13 DCFH-DA (a ROS probe) staining images of PA-stimulated INS-1 cells under different treatment conditions. Scale bar: 50 μm.

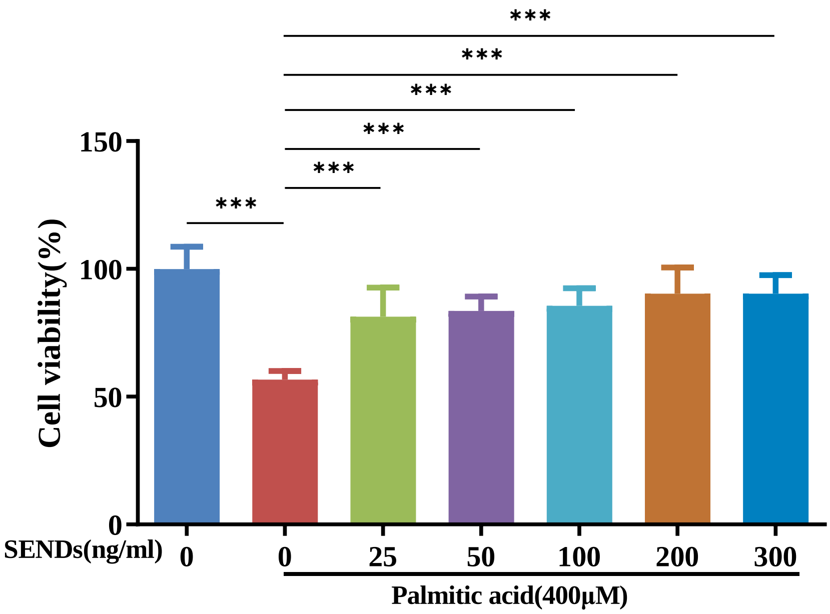

Figure S14 Cell viability of palmitic acid-stimulated INS-1 cells treated by SENDs at different concentrations. Data represent means ± S.D. from three independent replicates, $^{***}P$<0.001.

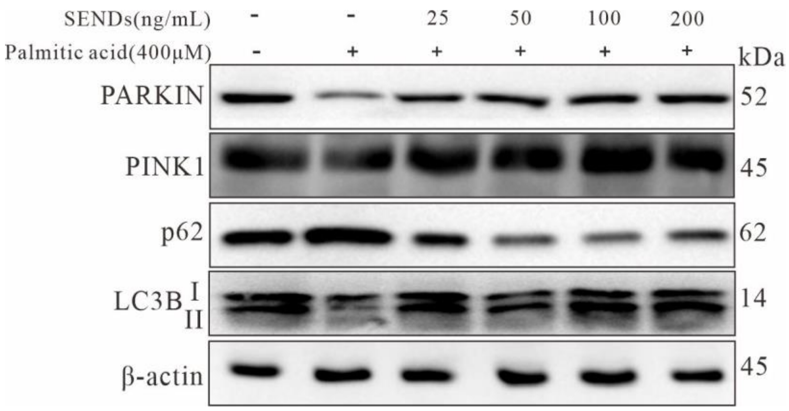

Figure S15 Western blot analysis of PARKIN, PINK1, p62 and LC3B II/I proteins expression in palmitic acid-stimulated INS-1 cells treated with different concentration of SENDs.

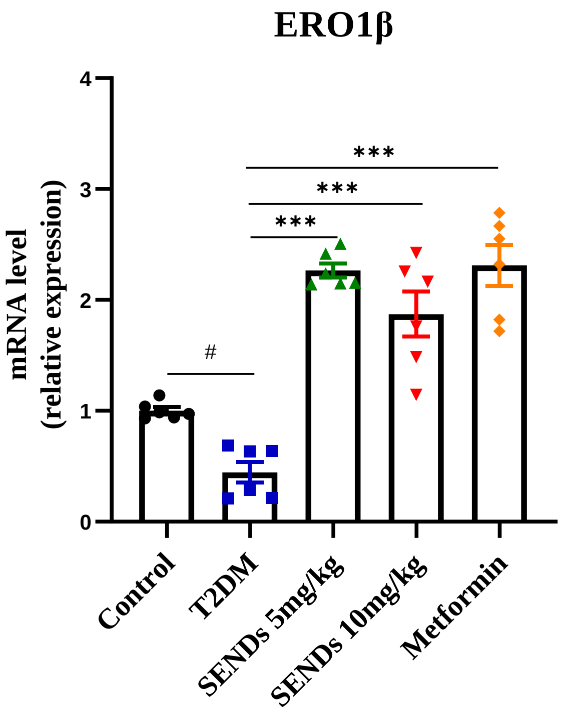

Figure S16 mRNA levels of ERO1β in pancreas tissue homogenates from each group. Data represent means ± S.D. from three independent replicates. ($^{\#}P$<0.05 *vs* Control group; $^{***}P$<0.001 *vs* T2DM group).

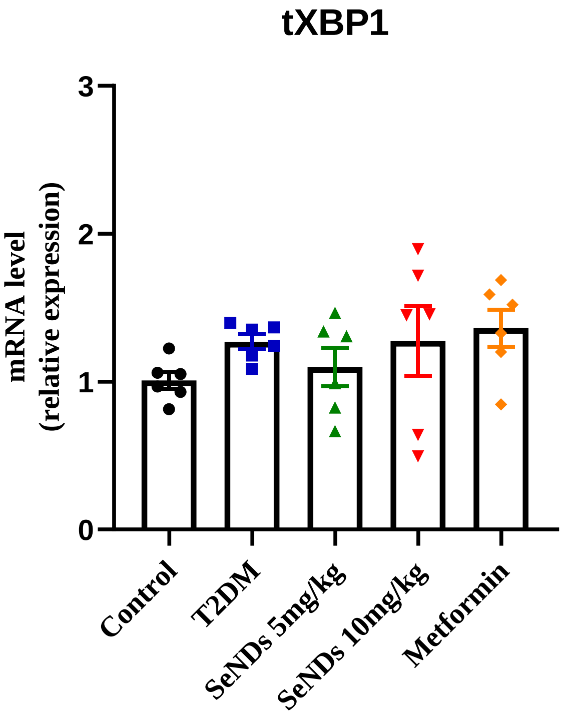

Figure S17 mRNA levels of tXBP1 in pancreas tissue homogenates from each group. Data represent means ± S.D. from three independent replicates.

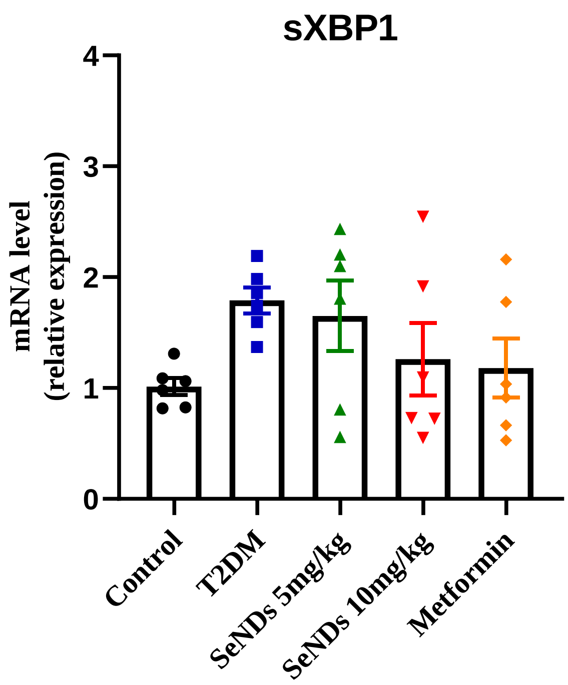

Figure S18 mRNA levels of sXBP1 in pancreas tissue homogenates from each group. Data represent means ± S.D. from three independent replicates.

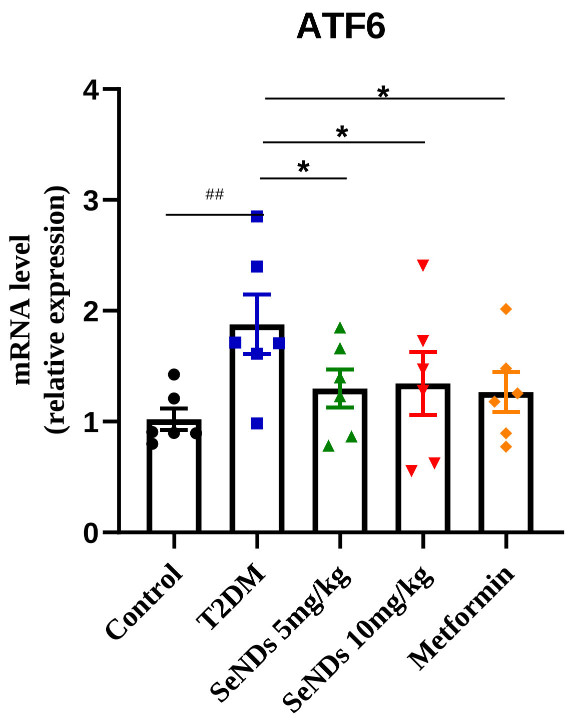

Figure S19 mRNA levels of ATF6 in pancreas tissue homogenates from each group. Data represent means ± S.D. from three independent replicates. ($^{\#\#}$*P*<0.01 *vs* Control group; **P*<0.05 *vs* T2DM group).

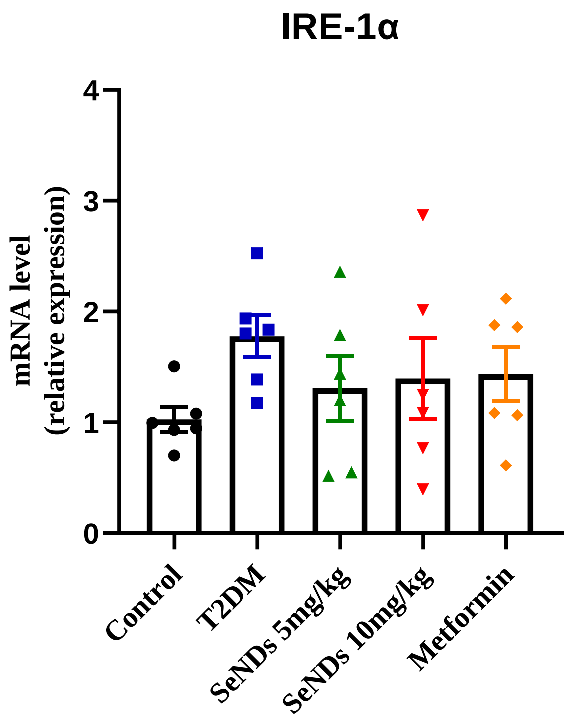

Figure S20 mRNA levels of IRE-1α in pancreas tissue homogenates from each group. Data represent means ± S.D. from three independent replicates.

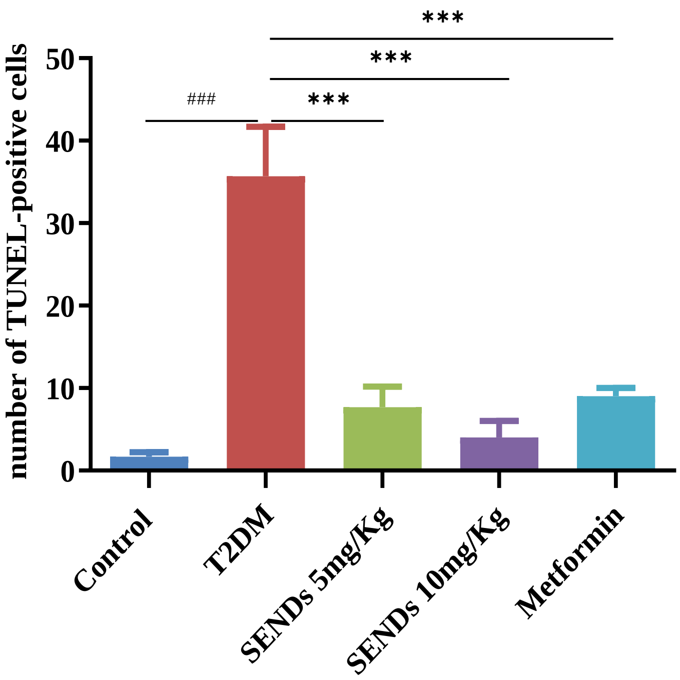

Figure S21 Quantification of TUNEL-positive cells in TUNEL and DAPI staining merge images of pancreatic tissues from each group. ($^{###}P$<0.001 *vs* Control group; $^{***}P$<0.001 *vs* T2DM group).
